# Molecular mechanisms of bamboo-derived miRNA-mediated gene regulation and dietary adaptation in giant pandas

**DOI:** 10.1186/s12864-025-12244-y

**Published:** 2025-11-19

**Authors:** Zheng Yan, Qin Xu, Xin He, Ying Yao, Dingzhen Liu, Hairui Wang

**Affiliations:** 1 Chengdu Research Base of Giant Panda Breeding, Chengdu, 610081 China; 2 The Conservation of Endangered Wildlife Key Laboratory of Sichuan Province, Chengdu, 610081 China; 3 Key Laboratory for Biodiversity and Ecological Engineering of Ministry of Education, Department of Ecology, College of Life Sciences, Beijing Normal University, Beijing, 100875 China

**Keywords:** Giant panda, Bamboo-derived microRNA, Exosome, Dietary adaptation, Gene regulation, Cross-kingdom RNA transfer, *HDAC9*, Metabolism, Immunity, Reproduction

## Abstract

**Background:**

Giant pandas subsist almost exclusively on bamboo, a low-nutrient, high-fiber plant. However, the molecular mechanisms underlying their dietary adaptation remain unclear. Recent evidence suggests that dietary plant-derived microRNAs (miRNAs) may influence gene regulation across species boundaries. This study aims to investigate the presence and functional significance of bamboo-derived miRNAs in giant pandas, and to explore their potential regulatory roles through gene expression modulation.

**Results:**

We successfully isolated and characterized plasma exosomes from giant pandas and identified 67 candidate bamboo-derived miRNAs by aligning small RNA sequences with bamboo shoot transcriptomes. Functional annotation revealed that these miRNAs target genes involved in metabolism, immunity, neurodevelopment, and cellular homeostasis. Among them, *HDAC9* was identified as a core gene targeted by multiple bamboo-derived miRNAs. Dual-luciferase reporter assays confirmed that two representative miRNAs, *miR166a* and *miR159*, directly bind to and suppress *HDAC9* 3’ untranslated region activity. Additionally, target enrichment analysis showed that these miRNAs may influence key signaling pathways, including MAPK and NF-kappa B. Several core target proteins, such as PRKACB, RAC2, and ADCY6, were implicated in inflammation, energy metabolism, and cardiovascular function. These findings suggest a broad and specific regulatory network mediated by dietary bamboo miRNAs.

**Conclusions:**

Our results demonstrate that bamboo-derived miRNAs are present in the blood of giant pandas and may modulate gene expression through cross-kingdom regulatory mechanisms. These miRNAs potentially contribute to dietary adaptation by regulating genes involved in metabolism, immune response, and reproductive processes. This study provides molecular insights into the unique plant-based diet of giant pandas and suggests that cross-kingdom RNA regulation may represent a widespread adaptive strategy across animal taxa.

**Supplementary Information:**

The online version contains supplementary material available at 10.1186/s12864-025-12244-y.

## Background

Dietary components serve not only as sources of energy and nutrients but also as potential modulators of gene expression across kingdoms. Bamboo, a unique member of the *Poaceae* family, exhibits highly specialized shoot development, and recent studies have suggested that microRNAs (miRNAs) may participate in regulating meristem function, morphogenesis, and nutrient metabolism in bamboo [1]. miRNAs share conserved biogenesis pathways and gene-silencing mechanisms in both plants and animals, primarily exerting regulatory effects through translational repression, mRNA cleavage, or degradation [2, 3]. In recent years, accumulating evidence has suggested that plant-derived miRNAs ingested through diet may influence endocrine function, metabolic homeostasis, and disease progression in animals [4, 5]. For instance, miRNAs such as miR159 from *Arabidopsis thaliana* have been detected in human and mouse [6], and shown to be inversely correlated with the incidence and severity of breast cancer, implying potential anti-tumor functions [6]. Further studies have demonstrated that these dietary miRNAs can circulate systemically and modulate gene expression in host tissues, indicating their potential for cross-kingdom regulatory activity [7]. Recent reviews have summarized the origin, stability, transport mechanisms, and functional roles of plant-derived miRNAs in mammalian systems, highlighting well-studied examples such as MIR168a, MIR156a, MIR159, and MIR2911, which illustrate the capacity of dietary miRNAs to regulate host gene expression across kingdoms [8]. However, since this hypothesis was first proposed in 2012 [7, 9], the stability, bioavailability, and functionality of dietary plant miRNAs have remained controversial. Some studies have questioned the authenticity of their origin and biological relevance [10, 11], and others have failed to detect plant miRNAs after dietary intake [12, 13], highlighting the urgent need for rigorous validation of their uptake, transport mechanisms, and regulatory roles. Current models propose that plant miRNAs may be absorbed by gastrointestinal epithelial cells and encapsulated in exosomes, thereby avoiding enzymatic degradation, and facilitating systemic transport to distal tissues [7, 14, 15]. Exosomes are nanoscale vesicles secreted by diverse cell types, present in various body fluids, and enriched with bioactive molecules such as proteins, lipids, mRNAs, and miRNAs [16, 17]. As novel messengers and effector molecules, exosomal miRNAs mediate intercellular communication and participate in development, immune responses, viral defense, organ function, and tumorigenesis [18]. Among biological fluids, plasma is considered an ideal source for exosomal miRNA extraction due to its high purity, stability, and yield [19–21], and has been widely applied in biomarker discovery and therapeutic target research in cancer. Therefore, investigating the exosome-mediated transport of plant-derived miRNAs holds promise for uncovering the molecular basis and biological significance of cross-kingdom regulation.

The giant panda (*Ailuropoda melanoleuca*) represents an extraordinary case of dietary specialization among mammals, as it relies almost exclusively on bamboo despite retaining a carnivorous digestive anatomy [22]. Over evolutionary time, pandas have undergone extensive adaptations to process this high‑fiber, low‑nutrient diet, including restructuring of gut microbiota, rewiring of metabolic pathways, and modulation of immune function, as demonstrated by multi‑omics studies [23–25]. Notably, pandas exhibit pronounced seasonal foraging behavior in spring and summer, during which they consume large quantities of nutrient‑rich bamboo shoots—a period that coincides with heightened reproductive activity and increased energy demands [26, 27]. This observation suggests that bioactive components in bamboo shoots, such as miRNAs, may influence gene expression to support reproduction, metabolism, and immune regulation. Indeed, plant miRNAs, especially those derived from edible tissues such as shoots, have been shown to survive digestion, enter the circulation, and retain biological activity in animal hosts [7, 28]. Although plant miRNAs have been detected in the blood or tissues of model organisms under controlled dietary conditions [29], their presence, targets, and regulatory roles in wild or endangered species remain largely unexplored.

To fill this critical knowledge gap, we conducted an integrative investigation of bamboo‑derived miRNAs in a natural dietary specialist. Specifically, we (1) isolated and sequenced small RNAs from giant panda plasma exosomes and from bamboo shoots, (2) assembled the bamboo shoot transcriptome de novo to serve as a reference, (3) identified candidate bamboo miRNAs by rigorous bioinformatic filtering, (4) predicted their host gene targets using complementary algorithms and performed Gene Ontology and KEGG enrichment analyses, and (5) experimentally validated selected miRNA–target interactions via qRT‑PCR and dual‑luciferase reporter assays. This comprehensive approach allows us to test the hypothesis that ingested plant miRNAs can modulate host gene networks and thus contribute to the unique dietary adaptation of the giant panda. By elucidating the cross‑kingdom mechanisms through which dietary small RNAs participate in metabolic, reproductive, and immune regulation, our study not only advances understanding of panda biology but also establishes a framework for exploring nutritional epigenomics and interspecies molecular communication in other organisms.

## Methods

### Subjects and sample collection

A total of six adult captive giant pandas, including three males (mean age = 12, SD = 4) and three females (mean age = 12, SD = 6) at the Chengdu Research Base of Giant Panda Breeding (CRBGPB) in Chengdu, Sichuan Province, China, were included in this study. After feeding on the bamboo shoots of *Pleioblastus amarus* for one month consecutively, we collected blood samples from each panda as well as samples of the bamboo shoots. The bamboo shoot samples originated from seasonal plants in Chengdu, Sichuan, China. The pandas had completed voluntary blood collection training and showed no signs of stress behavior during our blood collection procedures. All blood samples were drawn from the left forelimb of the pandas. Fresh blood samples were collected in 5 mL EDTA tubes (KANG JIAN, CHN), and plasma samples were separated and stored in 1.5 mL EP tubes (AXYGEN, USA) at −80 °C until further use. All animals were healthy during the experiment and were fed and cared for according to the standard animal husbandry procedures at CRBGPB.

### Extraction and morphological observation of exosomes

We extracted plasma exosomes by taking a volume of 250 μL of plasma sample and using the ExoQuick Plasma Kit (SBI, USA) following the manufacturer's instructions [30]. After the extraction, we took 10 μL of the exosome sample and added it onto a 200-mesh copper grid for 1 min. We gently removed the excess liquid by blotting the grid with filter paper. Next, we added 10 μL of uranyl acetate (phosphotungstic acid) onto the copper grid for 1 min. Again, we removed the excess liquid by blotting the grid with filter paper. We then air-dried the grid at room temperature for 5 min. Finally, we imaged the grid using a transmission electron microscope (Tecnai G2 spirit, Czech Republic) at an accelerating voltage of 80 kV.

To measure the size and concentration of plasma exosomes, we employed nanoparticle tracking analysis (NTA) with a ZetaView S/N 17–310 instrument (Particle Metrix, Meerbusch, Germany) and its associated software, ZetaView (v.8.04.02). Prior to analysis, isolated exosome samples were diluted with 1X PBS buffer (VivaCell, Shanghai) to achieve the requisite concentration for size and concentration measurements. We recorded and analyzed NTA measurements at 11 different positions and calibrated the ZetaView system using 100 nm polystyrene particles. Throughout the experiment, we maintained the temperature at approximately 26 °C to ensure consistency of results.

Transmission electron microscopy (TEM) revealed that the plasma exosomes exhibited the typical cup-shaped, double-layered vesicular morphology (Figure S1). NTA analysis showed that the mean diameter of the plasma exosomes was 126.6 ± 54.9 nm. The concentration of exosomes in the original plasma samples was estimated at 7.0 × 10^9^ particles/mL.

### RNA extraction and library construction

We used the miRNeasy Micro Kit (QIAGEN, Valencia, CA, USA) to extract plasma exosomal RNA samples. Subsequently, we assessed the integrity and quality of the total RNA extracted from exosomes and bamboo shoots using the Agilent Technologies 2100 Bioanalyzer. Next, we used the NEBNext Small RNA Library Prep Set for Illumina kit (NEB, USA) to construct small RNA libraries. In brief, RNA molecules were ligated with sequencing adapters on both ends, followed by reverse transcription into cDNA and PCR amplification. Subsequently, gel electrophoresis was performed to purify the PCR products in the size range of 140 to 160 bp for small RNA library preparation. After passing quality control on the Agilent 2100 Bioanalyzer, the libraries were subjected to downstream sequencing procedures.

### Small RNA sequencing and data preprocessing

We conducted small RNA sequencing on both giant panda plasma exosomes and bamboo shoot samples using the Illumina NovaSeq 6000 platform (San Diego, CA, USA) with the Solexa sequencing-by-synthesis method. Raw reads were processed using Cutadapt (v1.14) to remove adapter sequences, followed by filtering out reads shorter than 15 nt or longer than 41 nt. Quality control was performed using the FASTX-Toolkit (v0.0.13), retaining reads with a minimum base quality of Q20 in at least 80% of bases. Reads containing ambiguous bases (N) were removed using the NGS QC Toolkit (v2.3.3), resulting in high-quality clean reads for downstream analysis. We then quantified the number and abundance of unique clean reads, analyzed their length distribution, and assessed duplication rates.

To further ensure data quality and specificity, we aligned clean reads against the Rfam database (v10.0) using Bowtie [31] with an E-value cutoff ≤ 0.01, to annotate and remove sequences derived from rRNA, snRNA, tRNA, cis-regulatory elements, and other non-coding RNAs (Table S1). Reads passing Rfam filtering were then aligned to transcript sequences with zero mismatches allowed; sequences perfectly matching transcripts and longer than 26 nt were considered potential mRNA degradation products and removed. Reads between 15 and 26 nt, combined with reads that did not match transcripts, were retained for known and novel miRNA identification. Finally, to eliminate repetitive elements, the remaining sequences were aligned to the Repbase database using RepeatMasker, and identified repetitive sequences were excluded from further analysis [32].

### Bamboo shoot de novo assembly transcriptome analysis

Due to the absence of a reference genome for *Pleioblastus amarus*, we performed de novo assembly to reconstruct its transcriptome, facilitating the identification of bamboo-derived miRNAs in giant panda plasma exosomes.

Fresh bamboo shoots were peeled, cut, and immediately ground in liquid nitrogen to preserve RNA integrity and prevent degradation. Total RNA was extracted using TRIzol reagent (Invitrogen, USA) following the manufacturer’s protocol. RNA quality and integrity were assessed using an Agilent 2100 Bioanalyzer. Transcriptome libraries were prepared with the VAHTS Universal V6 RNA-seq Library Prep kit (Vazyme Biotech, China), and library quality control and sequencing procedures followed standard protocols.

Raw sequencing reads in fastq format were processed using Trimmomatic (v0.32) [33] to remove reads containing poly-N sequences and low-quality bases, yielding clean reads. Clean reads were assembled de novo using Trinity [34] with the paired-end assembly method. Among assembled transcripts, the longest isoform per gene was selected as the unigene for downstream analysis. Bowtie2 (v2.3.3.1, parameters: –reorder -k 30 -t) was used to calculate the number of reads from each sample that mapped to the unigenes, where –reorder ensures the output order matches the input order, -k 30 retains up to 30 valid alignments per read.

Functional annotation of unigenes was performed using Diamond (v2.0.15) [35] against six authoritative databases with a filtering threshold of –evalue 1e-5, including GO, KEGG, SwissProt, eggNOG, and NCBI NR.

### Identification of bamboo-derived miRNAs in plasma exosomes

We first aligned clean reads to the Rfam database (v.10.1) using Bowtie software to remove various non-coding RNAs, including rRNA, scRNA, small nuclear RNAs (snRNAs), small nucleolar RNAs (snoRNAs), and tRNAs. The remaining reads were then aligned to the giant panda reference genome, and all reads that mapped to the giant panda genome were discarded to eliminate endogenous sequences. The unmapped reads were subsequently aligned to the unigene sequences obtained from the bamboo shoot de novo transcriptome assembly. After filtering against Rfam, the transcriptome, and repetitive sequences, the remaining reads were annotated using Bowtie, aligned to plant miRNAs in the miRBase database (version 22.0) with zero mismatches to identify potential bamboo-derived miRNAs. Considering that giant pandas obtain over 99% of their diet from bamboo, these non-panda-derived miRNAs were presumed to originate from bamboo.

For bamboo shoot miRNA identification, we applied a similar approach. Clean reads from bamboo shoot small RNA sequencing were first aligned to bamboo shoot unigenes. After filtering, reads were mapped to known mature plant miRNA sequences in miRBase using Bowtie. Unannotated sequences were subjected to novel miRNA prediction with miRDeep2. RNA secondary structures of genome-mapped sequences were predicted using RNAfold. Sequences capable of forming typical miRNA hairpin precursors were considered as potential novel miRNAs. Finally, sequences of the potential bamboo-derived miRNAs were compared with identified bamboo shoot miRNAs without allowing mismatches. Those with perfect matches were confirmed as bamboo shoot-derived miRNAs.

We quantified miRNA expression levels using the transcripts per million (TPM) metric [36], calculated as:

$$\text{TPM}=\text{N}/\text{M}*{10}^{6}$$

where N is the number of reads mapped to a specific miRNA and M is the total number of mapped reads in the sample.

### Target gene prediction and functional analysis

We predicted target genes of bamboo and bamboo-derived miRNAs using bamboo unigenes and the giant panda genome as reference sequences, respectively. For bamboo shoot miRNAs, target prediction was performed with the GSTAr tool (v.1.0), using thresholds of MFEratio > 0.65 and AllenScore < 4 [37]. Using the conserved targets of well-characterized miRNA families to validate our predictions is a reliable strategy. To this end, we annotated the predicted transcripts by aligning the bamboo shoot transcript sequences against both the Swiss-Prot and NCBI NR protein databases using Diamond blastx, retaining only the top hit for each transcript. The output included transcript ID, database sequence ID, percentage identity, e-value, bit score, and protein description. We then filtered for reliable matches, keeping only transcripts with sequence identity greater than 30% and e-value less than 1e-5, and extracted the corresponding standard gene names from the protein descriptions. This approach allowed us to map the predicted transcripts to known gene names, thereby validating the effectiveness of our prediction strategy.

For giant panda target gene prediction, we applied two widely used algorithms, TargetScan (v.5.0) and miRanda (v.3.3a), using parameters appropriate for mammalian miRNA–mRNA interactions. Specifically, high complementarity is required primarily in the seed region, rather than along the entire sequence as in plants. Thresholds were set at TargetScan score ≥ 50 and Miranda energy ≤ −10. The final set of predicted target genes was defined as the intersection of results from both algorithms. Subsequently, Cytoscape software (v.3.10.1) [38] was employed to construct the miRNA-target gene regulatory network.

Functional annotation and enrichment analyses of miRNA target genes were performed using the Gene Ontology (GO) and Kyoto Encyclopedia of Genes and Genomes (KEGG) databases [39]. The enrichment factor was calculated by dividing the number of annotated genes involved in a specific biological process by the total number of annotated genes in the genome, quantifying the relative significance of the process within the genome [40].

### Protein network interaction analysis

We selected the top 100 predicted target genes and set the organism to Ailuropoda melanoleuca with a minimum required interaction score of 0.4. The expression patterns of bamboo-derived miRNA target genes in giant pandas were examined, and their relationships were assessed using protein–protein interaction (PPI) data from the STRING database (v.12.0, https://cn.string-db.org/, accessed on February 29, 2025). Disconnected nodes were excluded from visualization. The PPI network was constructed and visualized using Cytoscape software. Core proteins were identified based on centrality measures, including degree centrality and betweenness centrality, following established methods [41].

### Real-time quantitative PCR (qRT-PCR) detection

To validate the expression levels of bamboo-derived miRNAs in giant panda blood, we performed qRT-PCR using a combined approach integrating both poly(A) tailing and stem-loop methods, aiming to balance specificity, sensitivity, and throughput.

Total RNA was extracted using TRIzol reagent, and RNA concentration and purity were assessed by UV spectrophotometry. For the poly(A) tailing-based reverse transcription, we used the miRNA 1 st Strand cDNA Synthesis Kit (by tailing A) from Vazyme Biotech. This kit adds a poly(A) tail to the 3′ end of miRNAs via poly(A) polymerase, followed by reverse transcription with a universal primer containing a specific sequence. The tailing and reverse transcription steps were completed in a single-tube reaction containing 2 × miRNA RT Mix, HiScript miRNA Enzyme Mix, RNase inhibitor, and the Universal reverse Q primer for qPCR detection. The total reaction volume was 20 μL, prepared on ice with total RNA as the template. Reverse transcription was performed at 37 °C for 60 min, followed by enzyme inactivation at 85 °C for 5 min. The resulting cDNA was either used directly or diluted for subsequent qPCR.

qPCR was conducted using AceQ qPCR SYBR Green Master Mix in a total volume of 10 μL, including 2 μL of cDNA, 0.2 μL of primers, and 5 μL of Master Mix. The cycling program was: initial denaturation at 95 °C for 10 min, followed by 40 cycles of 95 °C for 10 s and 60 °C for 60 s. Melting curve analysis was performed to confirm amplification specificity (Supplementary file_Melt Curve Plot). Relative expression levels were calculated using the Delta-Delta Ct (2^−ΔΔCt^) method, with three technical replicates per sample. Calculations were performed in Microsoft Excel, and the average values were used for subsequent statistical analysis. miRNA-specific forward primers were designed based on the mature miRNA sequences, substituting RNA uracil (U) with thymine (T). The universal reverse primer provided in the kit (Tm ≈ 66 °C) was used consistently across all miRNA qPCR assays to ensure reproducibility and accuracy. To enhance primer thermostability and amplification efficiency, some miRNA forward primers were appended with a protective “TGCGC” sequence at the 5′ end, which optimizes primer-template binding and reduces primer-dimer formation, thereby improving sensitivity for low-abundance miRNAs. All primer sequences are listed in Table S2.

For secondary validation, bamboo-derived miRNAs detected in all giant panda blood samples were further confirmed by stem-loop RT-qPCR. Total RNA from serum samples was extracted using TRIzol reagent. Reverse transcription was performed using stem-loop primers specific to each miRNA, enabling high specificity suitable for distinguishing miRNA family members and precursors, and providing enhanced sensitivity for low-abundance targets. The RT reaction was conducted in two steps: first, genomic DNA contamination was eliminated by including specific loop primers; second, cDNA synthesis was performed using HiScript II Select qRT SuperMix II with the conditions of 50 °C for 15 min and 85 °C for 5 s to terminate the reaction.

qPCR employed AceQ qPCR SYBR Green Master Mix with a total volume of 10 μL. The amplification program comprised 95 °C for 10 min, followed by 40 cycles of 95 °C for 10 s and 60 °C for 60 s, with melting curve analysis to verify specificity. Each sample was run in triplicate, and cDNA was diluted threefold prior to qPCR. U6 small nuclear RNA served as the internal reference gene. Primers included specific forward primers based on mature miRNA sequences, stem-loop reverse transcription primers (loop primers), and a universal reverse primer (R primer: CCAGTGCAGGGTCCGAGGTATT). Detailed primer sequences are provided in Table S3.

### Dual-luciferase reporter assay

The binding sites of aly-miR166a and ahy-miR159 were selected based on the highest-scoring predictions from BiBiServ-hybrid. miRNA function mainly depends on the seed region (nucleotides 2–8 from the 5′ end), and perfect continuous base pairing is not required for repression [42, 43]. To verify the interaction between miRNAs (aly-miR166a, ahy-miR159) and the target gene *HDAC9*, the wild-type (WT) and mutant (MT) 3′-UTR fragments of *HDAC9* were cloned into the pmirGLO vector, positioned downstream of the Firefly luciferase gene. The regulatory effect was evaluated by the ratio of Firefly to Renilla luciferase activity.

One day prior to transfection, 293 T cells were seeded in 24-well plates to reach 60—70% confluence at the time of transfection. Cells were cultured in DMEM supplemented with 10% FBS. For each well, 1 μg of plasmid DNA (WT1: 0.3489 μg/μL; MT1: 0.3036 μg/μL; WT2: 0.5906 μg/μL; MT2: 0.2264 μg/μL) was diluted in 50 μL serum-free DMEM. miRNA mimics (final concentration 50 nM) were diluted in 50 μL serum-free DMEM. Separately, 4 μL Lipofectamine 2000 was diluted in 50 μL serum-free DMEM. The three components were gently mixed and incubated at room temperature for 15 min, then brought up to 500 μL with serum-free DMEM. After removing the original culture medium, the transfection mixture was added to each well, followed by incubation at 37 °C for 4—6 h. Subsequently, the medium was replaced with DMEM containing 10% FBS, and cells were cultured for an additional 48 h. Five biological replicates were included for each group. The transfection groups included: NC-mimics + *HDAC9*-WT1/WT2, aly-miR166a/ahy-miR159 mimics + *HDAC9*-WT1/WT2, and their corresponding MT controls.

The dual-luciferase assay was performed using the JKR23008 kit according to the manufacturer’s instructions. Briefly, 200 μL of cell lysis buffer (CLB) was added to each well and incubated on ice for 5 min. Lysates were centrifuged at 10,000 rpm for 5 min, and the supernatant was collected. Firefly (LRB + LRS) and Renilla (LRB II + LRS II) substrates were prepared at 1 × working concentration. For luminescence measurement, 10—20 μL of cell lysate supernatant and 100 μL of the corresponding substrate working solution were added into wells of a 96-well white plate. Luminescence was detected at 350–700 nm for 1 s using a plate reader. The relative luciferase activity was calculated as the ratio of Firefly RLU to Renilla RLU, reflecting the regulatory effect of miRNAs on *HDAC9* reporter gene expression.

### Statistical analysis

We applied the hypergeometric distribution test [44] to calculate *P* values for the significant enrichment of Gene Ontology (GO) terms and Kyoto Encyclopedia of Genes and Genomes (KEGG) pathways among the predicted miRNA target genes. To control for multiple testing, *P* values were adjusted using the Benjamini & Hochberg method to obtain the false discovery rate (FDR). GO terms and KEGG pathways with adjusted *P* values (FDR) ≤ 0.05 were considered significantly enriched. For the luciferase assays, each experimental group included five biological replicates, and differences between groups were analyzed using independent-samples t-tests. A *P* value < 0.05 was considered statistically significant.

## Results

### Functional annotation and miRNA target pathway analysis in bamboo shoots

**Fig. 1:**
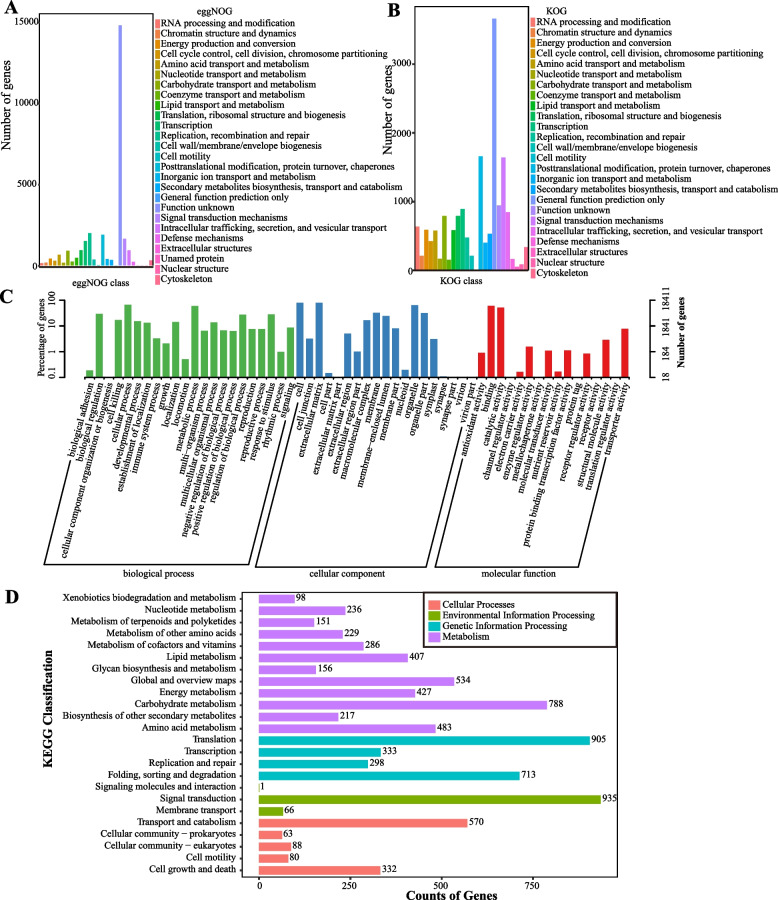
Functional annotation of bamboo shoot unigenes. **A** EggNOG classification of transcripts from the bamboo shoot de novo transcriptome assembly. The x-axis shows EggNOG functional categories; the y-axis indicates the number of annotated genes. **B** KOG classification of transcripts from the bamboo shoot de novo transcriptome assembly. The x-axis represents KOG functional categories; the y-axis shows the number of annotated genes. **C** GO classification of transcripts from the bamboo shoot de novo transcriptome assembly. The x-axis denotes GO categories; the left y-axis represents the proportion of annotated genes per category, and the right y-axis shows the total number of genes annotated to each category. **D** KEGG classification of transcripts from the bamboo shoot de novo transcriptome assembly. The x-axis indicates the number of annotated genes; the y-axis lists Level 2 pathway categories. Numbers at the end of each bar denote the count of genes assigned to the corresponding pathways

Transcriptome sequencing of bamboo shoots generated raw data with a Q30 score of 95.50% and an effective data volume of 11.86 Gb (Figure S2). The average GC content was 53.30%. De novo assembly yielded 43,006 unigenes (Table S4), totaling 39,572,834 bp with an average length of 920 bp. The mapping rate of reads to unigenes was 81.31%. Comprehensive annotation against multiple databases showed that 28,153 unigenes (65.46%) were annotated in the eggNOG database. The top functional categories included replication, recombination, and repair; posttranslational modification, protein turnover, chaperones; signal transduction mechanisms; and transcription (Fig. 1A). In the KOG database, 15,359 unigenes (35.71%) were annotated, primarily related to posttranslational modification, protein turnover, chaperones, signal transduction, and transcription (Fig. 1B). Gene Ontology (GO) annotation classified 18,411 unigenes (42.81%) mainly into cellular components (cell, cell part, organelle), biological processes (cellular process, metabolic process, biological regulation), and molecular functions (binding, catalytic activity) (Fig. 1C). Additionally, 9,251 unigenes (21.51%) were mapped to KEGG pathways, with predominant representation in signal transduction, translation, and carbohydrate metabolism (Fig. 1D).

**Fig. 2:**
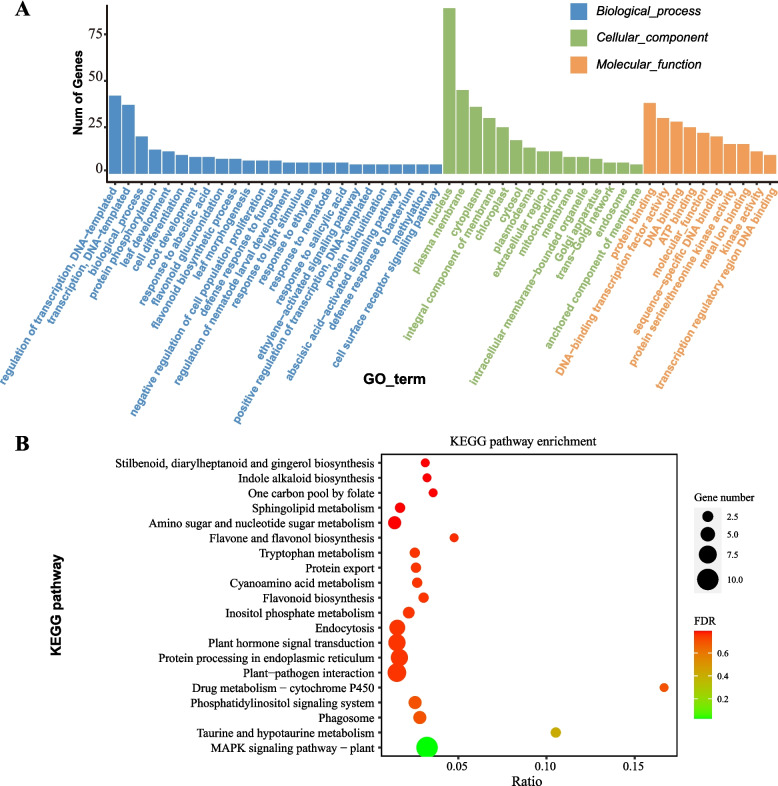
Functional annotation of target bamboo genes of bamboo shoot miRNAs. **A** Top 50 significantly enriched GO terms for genes targeted by bamboo shoot miRNAs. The y-axis shows the number of genes enriched in each GO term, while the x-axis lists the specific GO terms. **B** Top 20 significantly enriched KEGG pathways for genes targeted by bamboo shoot miRNAs

A total of 343 known miRNAs and 40 novel miRNAs were identified in bamboo shoot samples. These miRNAs were classified into 49 distinct families, with the MIR159 family being the most abundant, followed by MIR162 and MIR166, collectively accounting for 80.89% of the total miRNA abundance. The top 20 most abundant miRNAs constituted 89.45% of the total miRNA pool (Table S5). Among them, The top 20 most abundant miRNAs were predicted to target 11,107 bamboo genes (Table S6). Notably, the known conserved miRNA family–conserved target pairs, such as miR159- *GAMYB* [45], and miR162–*DCL1* [46], miR396–*GRFs* [47], and miR160–*ARFs* [48] were also identified in our bamboo shoot dataset, further supporting the reliability of our predictions. Functional annotation of these target genes revealed significant enrichment in 44 GO terms, which included key biological processes such as plant growth and development, transcriptional regulation, cell differentiation, photosynthesis-related light reactions, and various metabolic pathways (Fig. 2A). KEGG pathway analysis indicated significant enrichment of the plant-specific MAPK (Mitogen-Activated Protein Kinase) signaling pathway. Moreover, the largest number of target genes were associated with pathways related to plant–pathogen interaction, plant hormone signal transduction, and phosphatidylinositol signaling system, highlighting the critical regulatory roles of these miRNAs in plant physiological processes (Fig. 2B).

### Identification of bamboo-derived miRNAs in panda exosome and functional analysis of their target genes in panda

On average, plasma exosome samples from six giant pandas yielded 36,563,330 reads (SD: 5,797,816). After quality control and filtering, an average of 25,882,315 clean reads per sample (70.78% of total reads) were retained. Following further filtering, 159 candidate bamboo-derived miRNAs were identified. Comparison with bamboo miRNAs resulted in the confirmation of 67 bamboo-derived miRNAs present in giant panda plasma exosomes, which were classified into 17 miRNA families (Table S7). The five most abundant families were miR166, miR1919, miR396, miR168, and miR482.

**Fig. 3:**
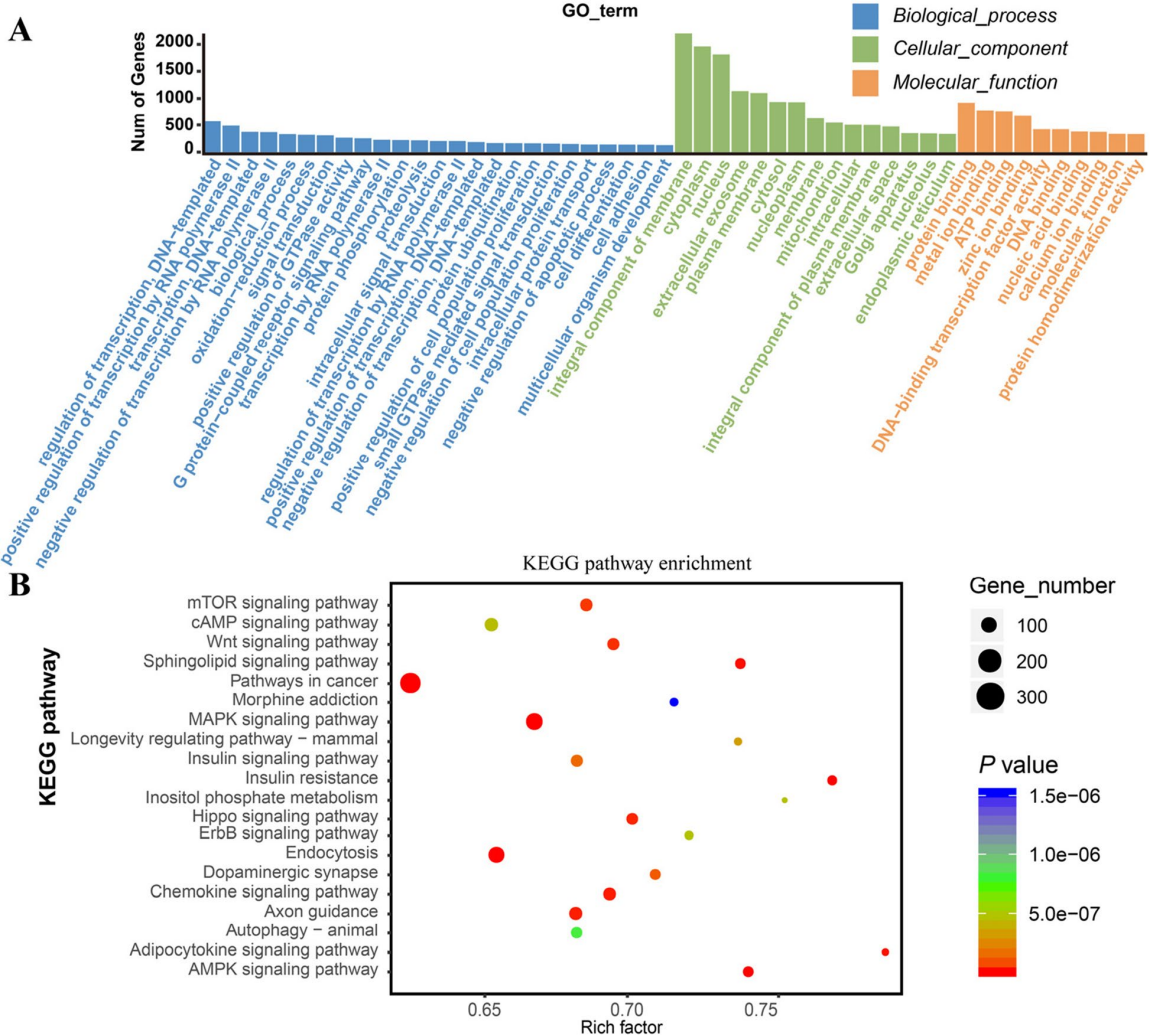
Functional analysis of giant panda genes targeted by bamboo-derived miRNA. **A** Top 10 enriched GO terms of biological process, cellular component, and molecular function identified in the GO enrichment analysis. **B** Bubble plot showing the top 20 enriched KEGG pathways among the target genes of potential bamboo-derived miRNAs detected in giant panda plasma

For the prediction of panda targets for bamboo miRNAs present in exosomes, it is assumed that once plant miRNAs enter the panda, they may function similarly to animal miRNAs. Therefore, the prediction was performed using animal miRNA parameters with two widely used algorithms, TargetScan (v.5.0) and miRanda (v.3.3a). Functional enrichment analysis of the 11,544 predicted target genes revealed 106 significantly enriched GO terms (FDR < 0.05). The largest number of genes were associated with cellular components, including integral component of membrane, cytoplasm, nucleus, plasma membrane, and extracellular exosome. Molecular functions enriched included protein binding, metal ion binding, and ATP binding. Among biological processes, prominent terms were G protein-coupled receptor signaling pathway, regulation of transcription (DNA-templated), and positive regulation of transcription by RNA polymerase II (Fig. 3A). Furthermore, KEGG pathway analysis identified significant enrichment in 105 pathways, including Pathways in cancer, PI3K-Akt signaling pathway, Human papillomavirus infection, MAPK signaling pathway, Endocytosis, Neuroactive ligand-receptor interaction, Ras signaling pathway, HTLV-I infection, Regulation of actin cytoskeleton, and Focal adhesion (Fig. 3B).

**Fig. 4:**
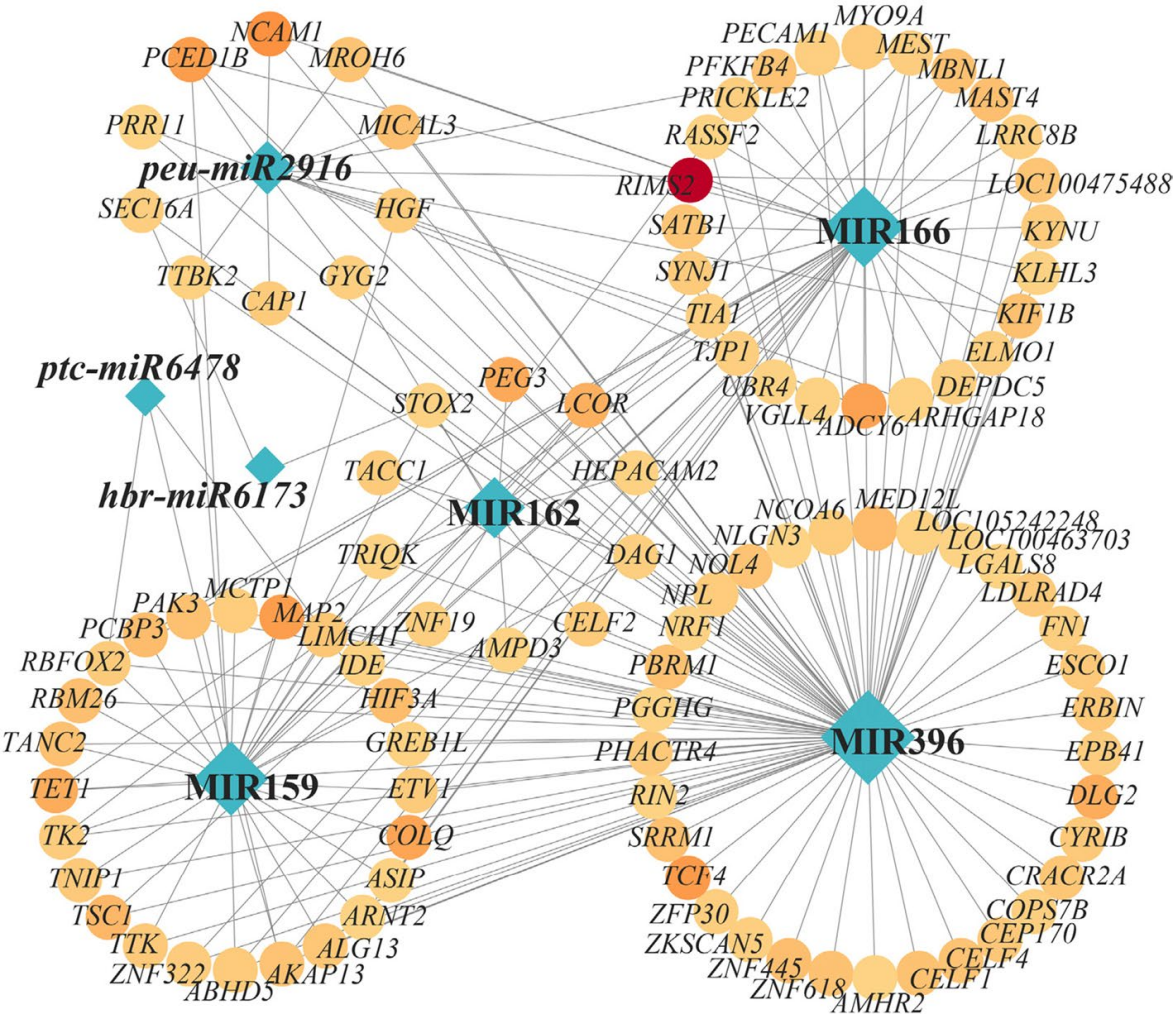
Regulatory network diagram of miRNAs and their target genes. Diamond-shaped nodes represent miRNA families, while circular nodes represent target genes. The darker the color of the circular nodes, the higher the predicted number of miRNAs targeting that gene. The size of both diamond and circular nodes corresponds to their degree value, with larger nodes indicating a greater number of connections

Among the 67 bamboo-derived miRNAs detected, 10 miRNAs were present in ≥ 50% of the six giant panda plasma samples (prevalence refers to the proportion of samples in which each miRNA was detected). Notably, two miRNAs were present in all samples: ptc-miR6478 (although not among the 10 most abundant miRNAs) and members of the miR166 family (including aly-miR166a-5p, bdi-miR166e-5p, csi-miR166e-5p, etc.). Using TargetScan and miRanda with parameters appropriate for mammalian miRNA–mRNA interactions, we identified a total of 11,544 shared target genes for the 67 bamboo-derived miRNAs in the giant panda genome. Regulatory network analysis focusing on miRNAs or miRNA families with prevalence ≥ 50% revealed that miR166 had the highest number of connections to target genes, followed by miR482 (Fig. 4). The top 10 target genes with the greatest number of regulatory connections were *RIMS2*, *DLG2*, *MAP2*, *MBNL1*, *LCOR*, *COLQ*, *NCAM1*, *PAK3*, *PCED1B*, and *ADCY6*.

### Core proteins identified by PPI network

**Fig. 5:**
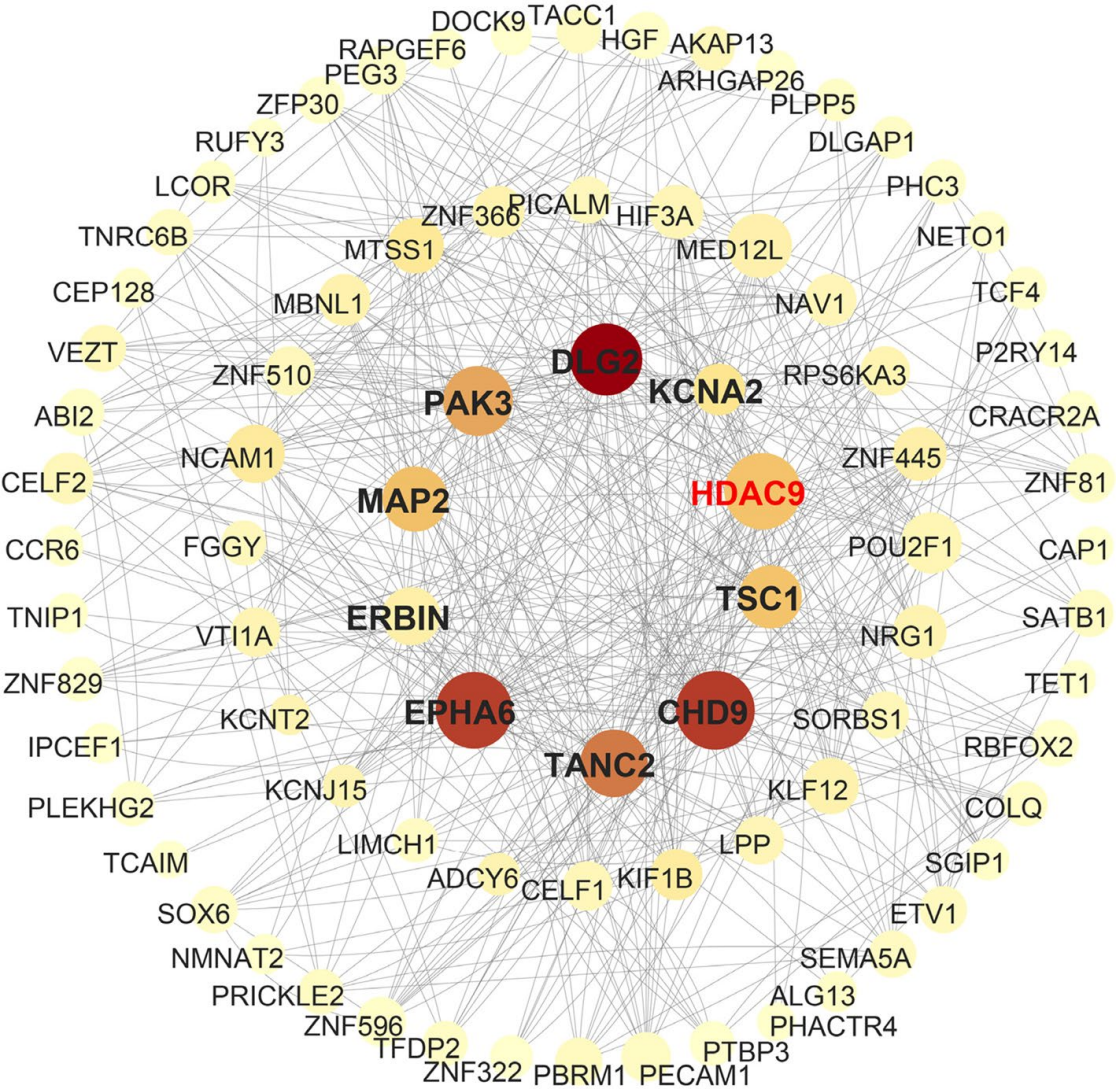
Protein–protein interaction network of mating-related genes. The node color intensity reflects betweenness centrality (BC) values, with darker nodes indicating higher BC. The size of each node corresponds to degree centrality, where larger nodes have more connections. PPI filtering was performed by retaining interactions with a minimum required interaction score of 0.9. The network shows significant enrichment with a PPI enrichment *P*-value < 0.001

Through protein–protein interaction (PPI) network analysis of bamboo-derived miRNA target genes, we identified ten core proteins—HDAC9, DLG2, CHD9, EPHA6, TANC2, PAK3, MAP2, TSC1, KCNA2, and ERBIN—based on betweenness centrality and degree centrality metrics (Fig. 5).

### qRT-PCR validation of bamboo-derived miRNAs in giant panda blood

We selected 26 bamboo-derived miRNAs with prevalence ≥ 50% and among the top 10 TPM values for validation using the poly(A) tailing qRT-PCR method. U6 snRNA was used as the internal reference, showing stable Ct values ranging from 23.09 to 23.61, indicating consistent template quality and amplification conditions. The Ct values of the target miRNAs ranged from 24.65 to 31.79, all within the reliable detection range. Relative expression levels of miRNAs were calculated using the Delta-Delta Ct (2^−ΔΔCt^) method, with sample 1 as the calibrator. ΔCt values (Ct_target—Ct_U6) were used as the basis for ΔΔCt calculation. This approach allows fold-change comparison of miRNA expression among samples. Results showed that most miRNAs had 2^−ΔΔCt^ average values > 0.4 in the sample 2, with the highest value reaching 9.32 *(sly-miR395a*), indicating accurate amplification and stable expression in blood (Table S8). All amplification and melting curves exhibited single, specific peaks without nonspecific products or primer dimers, fully demonstrating the specificity and sensitivity of the poly(A) tailing qRT-PCR method for detecting bamboo-derived miRNAs in giant panda blood. For further validation using the stem-loop qRT-PCR method, miRNAs with 100% prevalence and those selected for dual-luciferase assays *(ptc-miR6478*, *aly-miR166a-5p*, ahy-miR159, *aly-miR159b-3p*, and *aly-miR159c-3p*) were tested. U6 snRNA Ct values remained stable between 23.40 and 23.98. The Ct values of the tested miRNAs ranged within detectable thresholds, their ΔCt values indicating efficient amplification (Table S9). The 2^−ΔΔCt^ relative expression values were close to or exceeded 0.7, further confirming the specificity and stable amplification of these miRNAs using the stem-loop qRT-PCR method. Melting curves presented single peaks without nonspecific amplification or primer dimer formation, demonstrating the true presence of these miRNAs in giant panda blood samples, and validating the specificity and sensitivity of the stem-loop qRT-PCR approach.

### Dual-luciferase reporter assay for *HDAC9* target validation

To validate the interactions between selected bamboo-derived miRNAs and the target gene *HDAC9*, we performed dual-luciferase reporter assays. Briefly, the wild-type (WT) and mutated (MT) 3′-UTR fragments of *HDAC9* were cloned into the pmirGLO reporter vector. 293 T cells were transfected with these constructs along with the corresponding miRNA mimics. Firefly and Renilla luciferase activities were measured 48 h post-transfection, and the relative luciferase activity (Firefly/Renilla ratio) was calculated to assess the regulatory effect of the miRNAs.

**Fig. 6:**
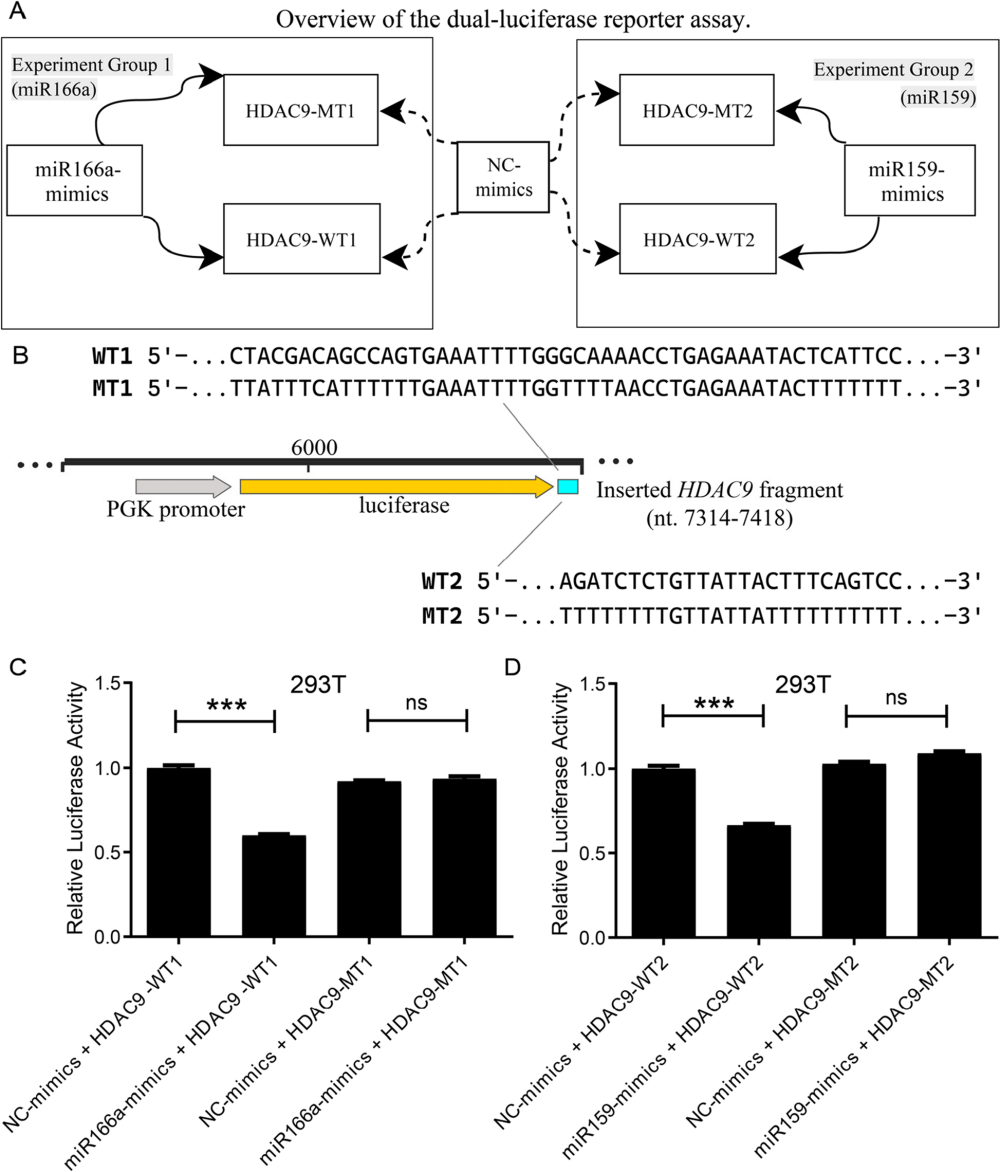
Dual-luciferase reporter assays validating the regulatory interaction between miR166a/miR159 and the 3′UTR of *HDAC9*. **A** Experimental design of the dual-luciferase reporter assay. **B** Schematic diagram of the dual-luciferase reporter constructs. The reporter cassette contains the PGK promoter driving luc2 luciferase, followed by the inserted HDAC9 fragment containing predicted miRNA binding sites. The exact nucleotide sequences of each site are shown, with the full sequences provided in Table S11. 293 T cells were co-transfected with (**C**) miR166a-mimics or (**D**) miR159-mimics along with wild-type (WT1/WT2) or mutant (MT1/MT2) *HDAC9* 3′UTR reporter constructs as experimental groups. Control groups were transfected with NC-mimics (negative control) plus the corresponding *HDAC9* 3′UTR constructs. *** *P* < 0.001. The predicted binding sites of aly-miR166a-5p and ahy-miR159 on *HDAC9* mRNA are shown in Figures S2 and S3

Given that multiple core bamboo-derived miRNAs target the gene *HDAC9* (Table S10), we selected *aly-miR166a* and *ahy-miR159* to validate their interactions with *HDAC9*. The experimental design of the dual-luciferase reporter assay is shown in Fig. 6A. A simplified schematic of the luciferase expression cassette, highlighting the inserted *HDAC9* fragment containing the predicted miRNA binding sites, including wild-type (WT1/WT2) and mutant (MT1/MT2) sequences, is shown in Fig. 6B. The exact nucleotide sequences of each WT and MT site are provided in Table S11. The predicted binding sites of aly-miR166a-5p and ahy-miR159 on *HDAC9* mRNA are shown in Figures S3 and S4.

In five biological replicates, we first measured the Firefly/Renilla luminescence ratio per well and normalized the values against the mean ratio of the NC-mimics + *HDAC9*-WT group (set as 1). The results showed that the normalized luciferase activity in the aly-miR166a-mimics + *HDAC9*-WT1 group was significantly decreased (*P* < 0.001). In contrast, in the mutated *HDAC9*-MT1 group, the normalized activity of aly-miR166a-mimics did not differ significantly from the NC-mimics + MT1 group (*P* > 0.05), indicating loss of miRNA repression due to seed region mutation (Fig. 6C, Table S12). Similarly, the normalized luciferase activity in the ahy-miR159-mimics + *HDAC9*-WT2 group was significantly lower than that of the WT2 control (*P* < 0.001), whereas no significant difference was observed between the ahy-miR159-mimics and the MT2 control in the *HDAC9*-MT2 group (*P* > 0.05) (Fig. 6D, Table S12). These results demonstrate that aly-miR166a and ahy-miR159 specifically bind to the wild-type 3’-UTR sequences of *HDAC9* and suppress reporter gene expression, while mutations in the seed regions (MT1, MT2) abolish miRNA binding and the associated repression. This confirms the specificity of aly-miR166a and ahy-miR159 binding to the *HDAC9* 3′-UTR in the in vitro luciferase assay.

## Discussion

The present study systematically analyzed bamboo-derived miRNAs present in giant panda plasma exosomes, providing molecular evidence that such dietary exogenous miRNAs may exist in circulation and potentially participate in host gene regulation. We successfully isolated exosomes from giant panda plasma, characterized by abundant circular or elliptical membrane vesicles with bilayer structures. Subsequent extraction of miRNAs from these exosomes, combined with alignment to de novo transcriptome sequencing data from bamboo shoots, led to the identification of 67 candidate bamboo-derived miRNAs. Target gene prediction indicated that these miRNAs primarily associate with five major panda gene families, with clear expression patterns and protein–protein interaction networks of core target genes delineated. Notably, multiple core bamboo-derived miRNAs jointly target the key gene *HDAC9*, suggesting its significant role in regulating gene expression in giant pandas. These findings provide compelling evidence for the potential cross-kingdom epigenetic influence of dietary miRNAs on mammalian gene expression. Importantly, this study provides novel molecular insights into the dietary specialization of giant pandas and establishes a foundation for further exploring the regulatory roles of exogenous miRNAs in wildlife nutrition and conservation biology.

Among the bamboo-derived miRNAs detected in giant panda plasma exosomes, members of the miR166, miR1919, miR396, miR168, and miR482 families were particularly enriched. These families are well-known regulators of plant development, stress responses, and disease resistance. For example, miR166, one of the most evolutionarily conserved plant miRNA families, has been shown to regulate genes associated with vascular tissue differentiation in bamboo and other terrestrial plants [49–51]. MiR396 modulates the expression of growth-regulating factors (GRFs), with its overexpression suppressing leaf cell proliferation, while its inhibition has been shown to enhance yield-related traits in rice by promoting the development of auxiliary branches and spikelets through GRF6 induction [52, 53]. Additionally, miR482 targets nucleotide-binding site leucine-rich repeat (NBS-LRR) genes, which are essential for plant disease resistance, partly through its role in generating secondary phased siRNAs (phasiRNAs) [54]. Although these miRNAs primarily function in plants, their stable presence in the host bloodstream raises the possibility that they may also participate in cross-kingdom regulatory processes, as suggested by previous reports on dietary miRNAs in other species [55]. Edible plant-derived exosome-like nanoparticles (ELNs) have been shown to enrich specific miRNAs, such as members of the miR166 and miR396 families, and predicted cross-kingdom target genes indicate potential regulation of human digestive, immune, and infection-related pathways [56]. Additionally, plant-derived MIR168a can enter animal serum and tissues through dietary intake and modulate mammalian physiological functions via its target gene *LDLRAP1* [7]. These findings highlight the need for further research into the bioactivity of dietary plant miRNAs and their potential applications in nutrition and disease modulation across species.

Notably, among the bamboo-derived miRNAs significantly enriched in giant panda plasma, only miR166 is relatively abundant in bamboo shoots, whereas others are not within the top ten most abundant miRNAs. Several factors may account for this discrepancy. First, the miRNA expression profile in bamboo shoots varies considerably across tissue types and developmental stages, with certain miRNAs enriched in specific tissues or stages but not ranking among the top ten in the whole shoot sample [8, 57]. Second, differences in miRNA stability may contribute, as miRNAs with 2’-O-methylated 3’ ends, high GC content, or encapsulation within exosomes or extracellular vesicles (EVs) are more likely to survive the transfer into the bloodstream and be detected [8, 58, 59]. Third, selective absorption or degradation during cross-kingdom transport may make some miRNAs more likely than others to enter the circulation [8, 58]. Together, these findings suggest that the profile of bamboo-derived miRNAs in giant panda plasma is influenced not only by their abundance in bamboo shoots but also by their stability, functional roles, and mechanisms of absorption or vesicle-mediated transport. Future studies examining the expression of these miRNAs across different tissues and developmental stages of bamboo, as well as their stability following ingestion by pandas, will help clarify their functional roles in the host.

Functional annotation of the candidate target genes regulated by the top 10 bamboo-derived miRNA families revealed their involvement in essential biological processes such as neuronal development, cytoskeletal organization, metabolic regulation, intracellular transport, and membrane signaling. Notably, the solute carrier family 26 member 6 (*SLC26A6*), a target of miR166, is implicated in mineral absorption, while collagen type V alpha 3 chain (*COL5A3*) participates in protein digestion and absorption. miR396 targets *RAB5C*, which plays a role in endocytosis, collectively highlighting the relevance of these genes to digestion and nutrient metabolism. As miRNAs often regulate multiple targets and vice versa, this indicates a complex and multilayered regulatory network [60]. KEGG pathway enrichment further showed significant involvement of bamboo-derived miRNA targets in 11 key signaling pathways. Among them, the MAPK cascade regulates cell proliferation, differentiation, apoptosis, and stress responses [61], with p38 MAPK specifically involved in germ cell proliferation and testicular development [62]. Additionally, pathways such as NF-kappa B signaling [63], rheumatoid arthritis [64], and viral protein interactions with cytokines suggest that these miRNAs may also influence immune regulation and inflammatory responses.

We identified ten core proteins potentially targeted by bamboo-derived miRNAs, many of which are closely linked to immune regulation, cancer development, and metabolic health. For instance, abnormal expression of *PRKACB* can suppress the PKA/CREB pathway, promoting tau hyperphosphorylation and brain aging [65], and is associated with colorectal and pancreatic cancers [66, 67]. *Rac2* plays an essential role in T cell activation and cytoskeletal remodeling [68–70], while ENPP1 is implicated in immune disorders and cancer [71]. *CAMK2D* is linked to heart disease [72], and *MAPK9/10* are involved in inflammation, apoptosis, and proliferation [73–75]. GMPS regulates chromatin dynamics and is overexpressed in several cancers [76, 77]. These findings suggest that bamboo-derived miRNAs may support host defense against diseases like arthritis, colorectal cancer, and pancreatic cancer. While direct experimental validation of cross-kingdom miRNA transfer in pandas remains challenging due to ethical and logistical limitations, the health impact of bamboo diet is supported by long-term field and feeding studies. Captive pandas are now typically fed mixed bamboo species [78], highlighting the unique value of this study under natural feeding scenarios. Bamboo shoots, rich in bioactive compounds, exhibit lipid-lowering, anti-diabetic, and prebiotic effects, and their consumption has been associated with improved gut microbiota and reduced fatty liver risk in pandas [22, 79]. Importantly, in wild giant pandas, bamboo shoot intake peaks in spring, coinciding with the breeding season [80]. However, under captive conditions, male pandas generally exhibit poor natural mating ability, which negatively impacts reproduction and the maintenance of genetic diversity [81, 82]. Our findings suggest a potential strategy to enhance male reproductive performance by promoting testicular cell proliferation. Furthermore, our study indicates that bamboo-derived miRNAs may contribute positively to both reproductive and immune health, providing molecular evidence for dietary adaptation and supporting the strategic provision of bamboo shoots in conservation and captive management practices.

In this study, *HDAC9* was identified as a shared target of multiple core bamboo-derived miRNAs and may play a pivotal role in the molecular adaptation of giant pandas to bamboo shoot consumption. *HDAC9* regulates downstream gene expression by modulating chromatin conformation and transcription factor activity, participating in complex disease mechanisms including cardiovascular disorders, osteoporosis, obesity, diabetes-related complications, liver fibrosis, and various cancers [83]. For instance, *HDAC9* promotes endothelial-to-mesenchymal transition (EndMT), accelerating atherosclerosis progression [84], and contributes to diabetic retinopathy by regulating *ANXA2*-mediated EndoMT [85]. Moreover, *HDAC9* plays important roles in maintaining vascular smooth muscle and endothelial cell function, with its inhibition showing therapeutic potential in cardiovascular diseases [86]. While the direct effects of *HDAC9* modulation in giant pandas remain to be experimentally verified, its regulation by bamboo-derived miRNAs suggests a potential influence on physiological processes such as vascular function and metabolic regulation. This highlights a novel cross-kingdom gene regulatory mechanism and emphasizes the possible relevance of dietary small RNAs in shaping host gene expression. These findings provide insight into how bamboo consumption might contribute to dietary adaptation and nutrition-immunity interplay in giant pandas. Beyond *HDAC9*, other core target genes of bamboo-derived miRNAs may contribute to metabolic regulation, neurodevelopment, and cellular homeostasis. For example, the transcriptional corepressor LCoR negatively regulates adipogenesis via interaction with C/EBP and chromatin remodeling, indicating a role in lipid metabolism [87]; *ADCY6*, a key enzyme in the cAMP signaling pathway, is involved in hepatic glucose production and β-adrenergic receptor–mediated heart failure, implicating it in energy metabolism and cardiac function [88, 89]; *PAK3* exhibits tumor-suppressive properties by promoting neuronal differentiation and inhibiting glioma progression [90]; the postsynaptic scaffold protein *TANC2* is associated with autism spectrum disorder and regulates neuronal excitation/inhibition balance and social behavior [91]; and *DLG2*, a candidate tumor suppressor gene, inhibits neuroblastoma cell proliferation by delaying cell cycle progression, with reduced expression linked to poor prognosis potentially due to *MYCN* amplification or 11q deletion [92]. These findings indicate that bamboo-derived miRNAs regulate multiple key genes across metabolic, immune, and neural pathways in giant pandas. This study enhances our understanding of dietary RNA-mediated gene regulation in a dietary specialist and highlights the broader potential for cross-kingdom regulation in other species, offering new insights into diet-host interactions, evolutionary adaptation, and RNA-based therapeutic development.

This study has several limitations. First, the prediction of miRNA target genes primarily relies on computational methods, which may produce false positives or false negatives, and without experimental validation, the functional roles of the predicted targets in biological processes cannot be fully confirmed. Second, miRNA expression patterns can vary across different tissues and developmental stages, which were not comprehensively covered in this study. Third, regarding the comparison of bamboo-derived and panda-derived miRNA abundance, all reads mapping to the giant panda genome were excluded when identifying bamboo miRNAs, preventing the acquisition of a complete panda miRNA dataset within the same analytical framework. Meanwhile, panda plasma miRNAs were analyzed separately, and their expression levels were normalized using transcripts per million (TPM), making direct comparisons of relative abundance between bamboo- and panda-derived miRNAs difficult. Therefore, a complete comparison between these two groups of miRNAs could not be provided at this stage. Future studies could incorporate both bamboo- and panda-derived miRNAs within a unified analytical framework, apply consistent quantification methods, and integrate multi-omics data, including transcriptomics, proteomics, and metabolomics, to further elucidate the functional roles of miRNAs and their target genes across tissues, developmental stages, and species.

## Conclusions

In conclusion, this study provides compelling evidence that bamboo-derived miRNAs are present in the plasma exosomes of giant pandas and likely contribute to physiological adaptation to a bamboo-based diet through cross-kingdom gene regulation. The identification of key target genes, including *HDAC9*, reveals potential molecular mechanisms linking dietary components to metabolic, immune, and neural pathways. These findings not only deepen the understanding of dietary specialization in giant pandas but also open new avenues for exploring the effects of dietary RNA in other species. Future work focusing on functional validation and mechanistic studies will be critical to fully elucidate the role of exogenous miRNAs in host biology and their potential applications in conservation and medicine.

## Supplementary Information

Supplementary Material 1.

Supplementary Material 2.

Supplementary Material 3.

Supplementary Material 4.

## Data Availability

The small RNA sequences of giant pandas and de novo RNA sequences of bamboo shoots datasets generated in this study have been uploaded to the NCBI sequence read archive (SRA) with a BioProject ID of PRJNA1040462.
