# Supplementary figures and images for: Molecular mechanisms of bamboo-derived miRNA-mediated gene regulation and dietary adaptation in giant pandas
Source: BMC Genomics. 2025 Nov 19;26:1062. doi: 10.1186/s12864-025-12244-y (PMC12628529; doi:10.1186/s12864-025-12244-y)

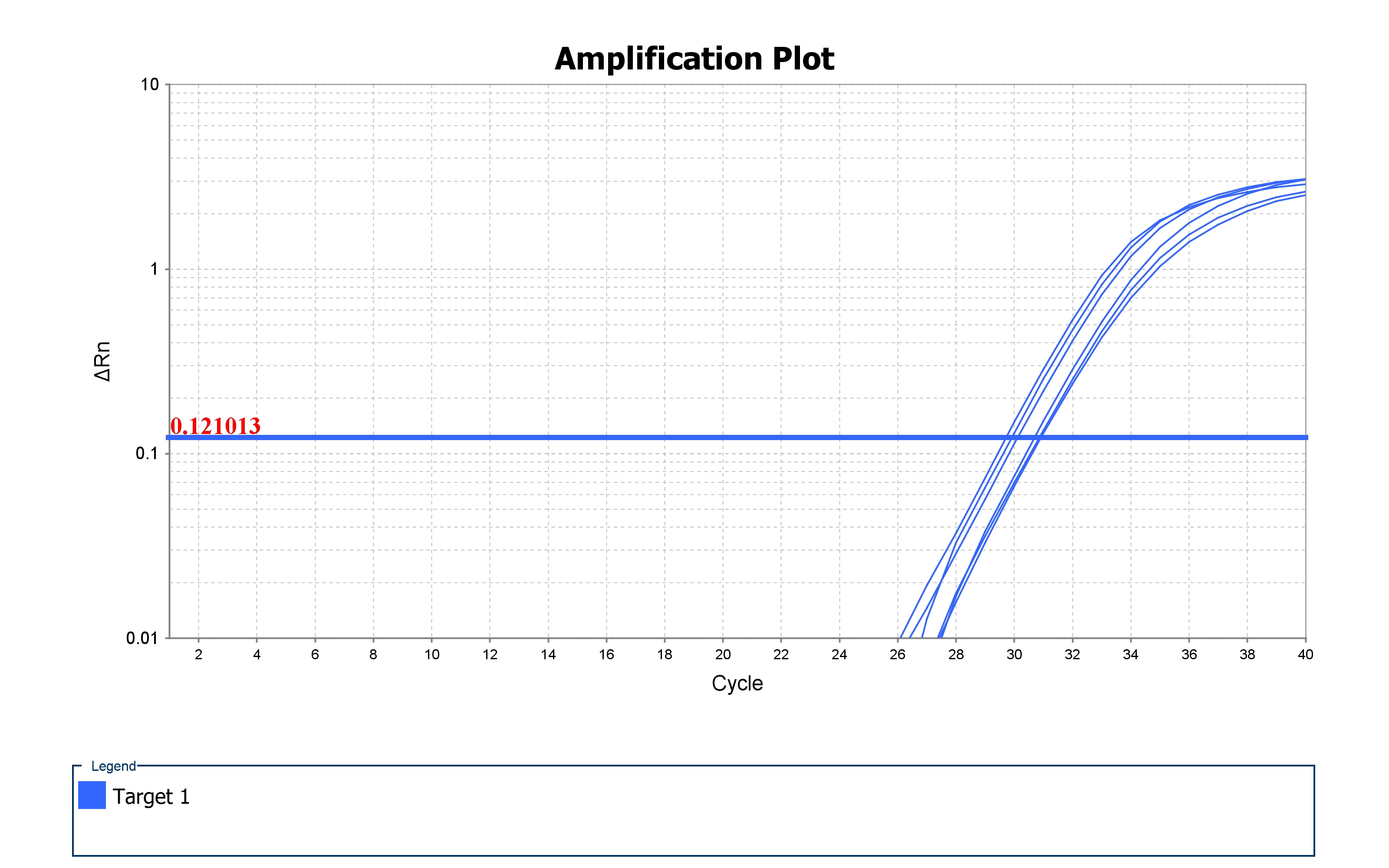

Supplement: Supplementary file 2 — Supplementary Material 2. [file 12864_2025_12244_MOESM2_ESM.zip › Supplementary file2-Amplification Plot/poly(A) tailing/aau-miR162.jpg]

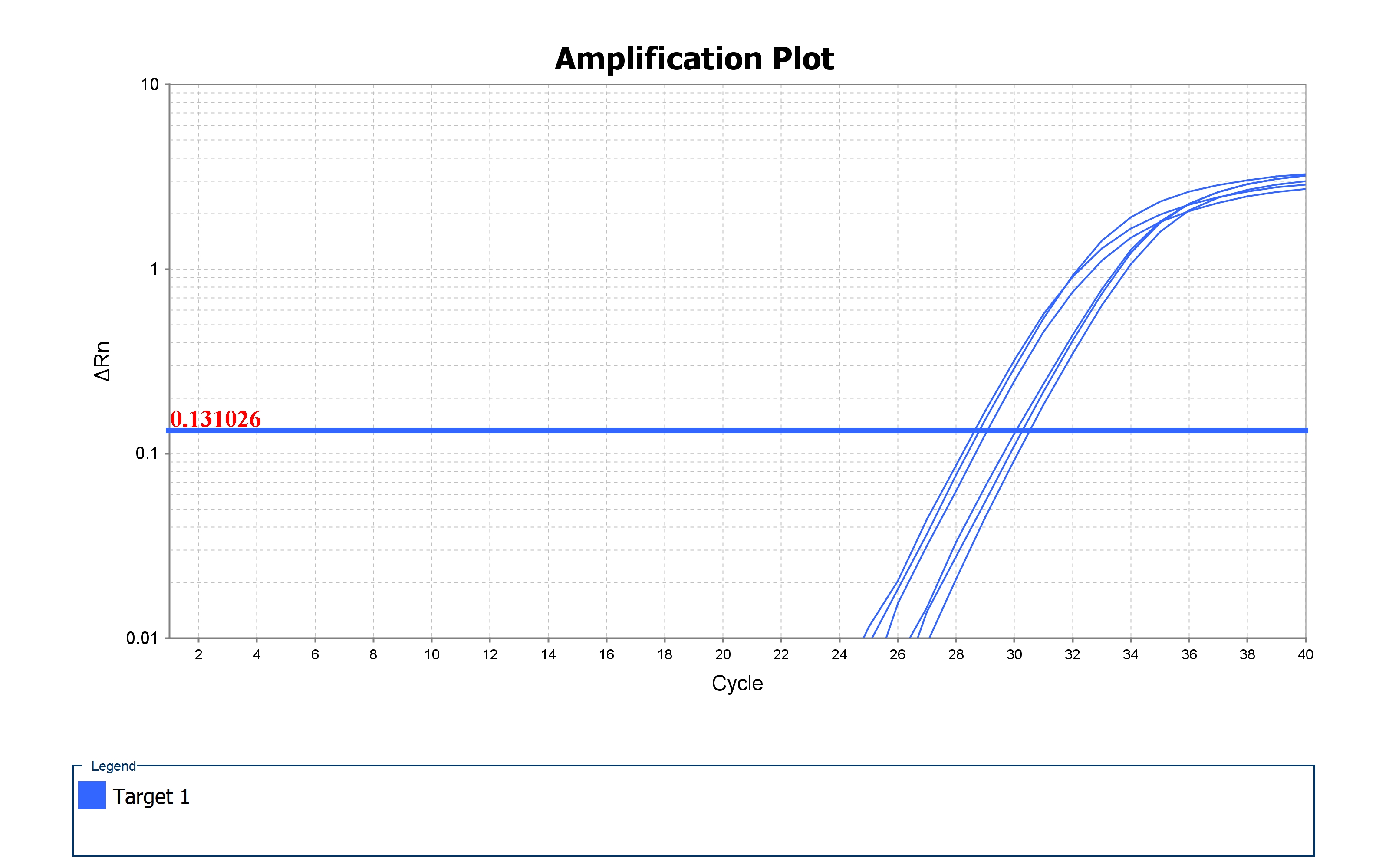

Supplement: Supplementary file 2 — Supplementary Material 2. [file 12864_2025_12244_MOESM2_ESM.zip › Supplementary file2-Amplification Plot/poly(A) tailing/aau-miR396.jpg]

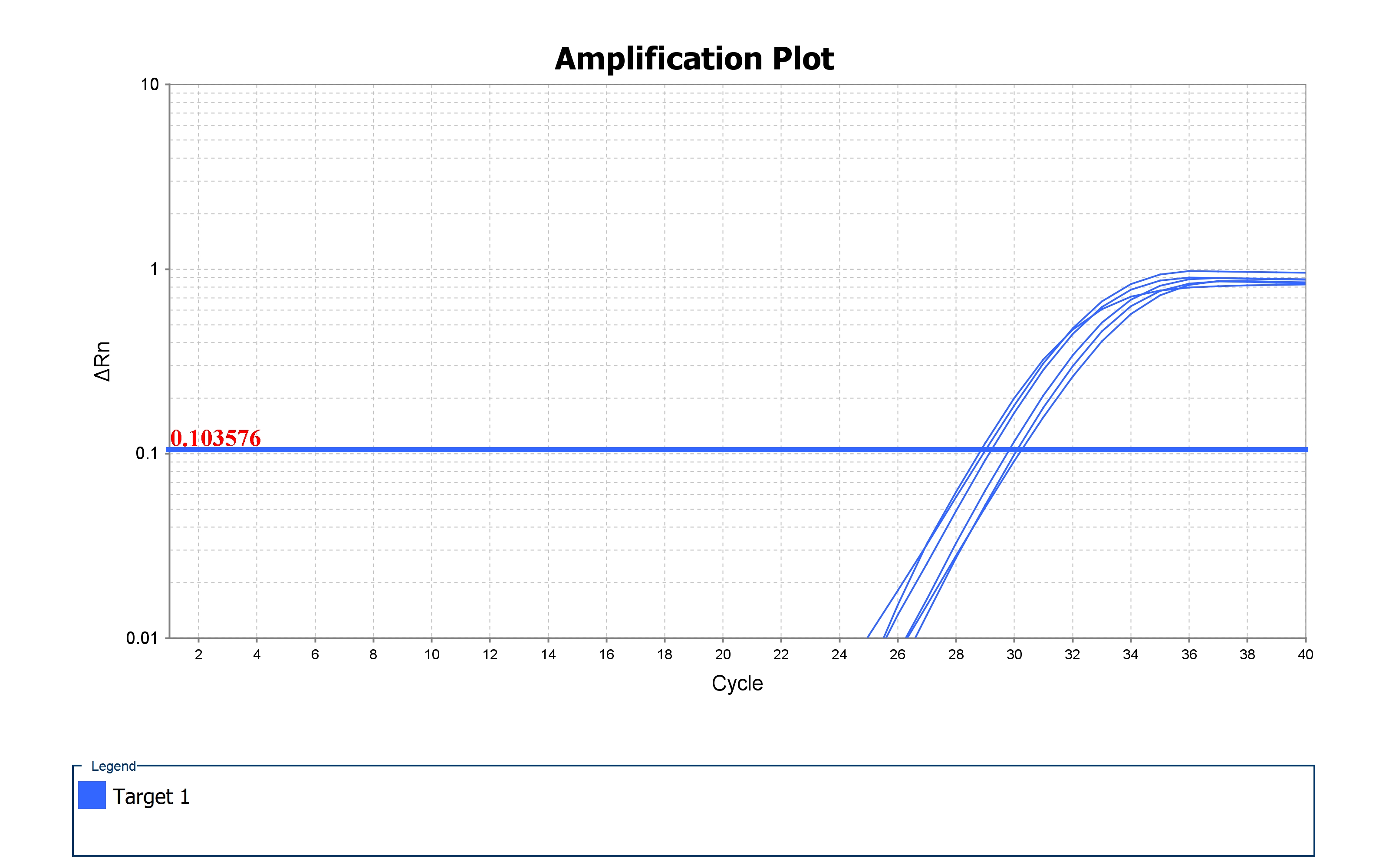

Supplement: Supplementary file 2 — Supplementary Material 2. [file 12864_2025_12244_MOESM2_ESM.zip › Supplementary file2-Amplification Plot/poly(A) tailing/ahy-miR159.jpg]

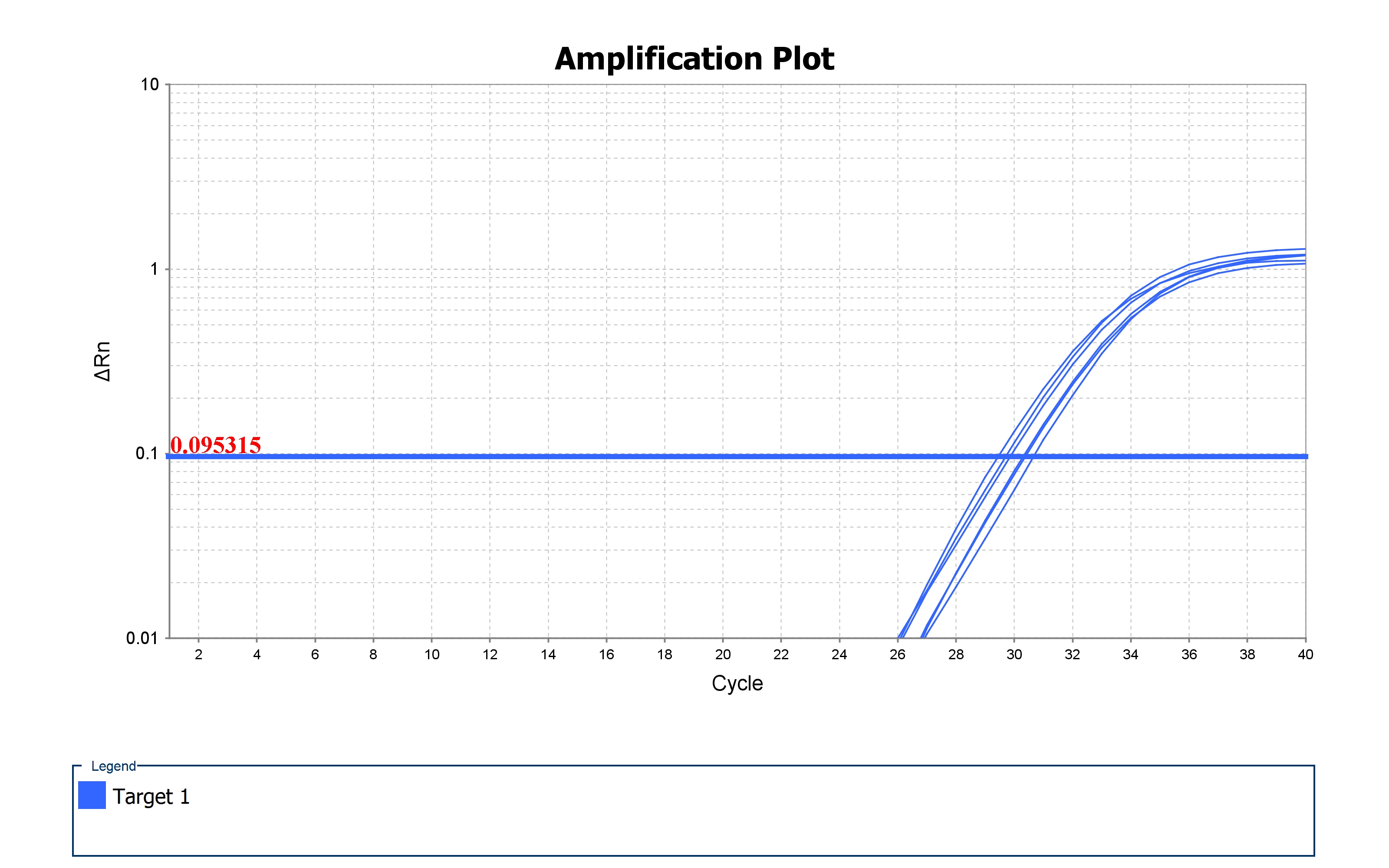

Supplement: Supplementary file 2 — Supplementary Material 2. [file 12864_2025_12244_MOESM2_ESM.zip › Supplementary file2-Amplification Plot/poly(A) tailing/aly-miR159b-3p.jpg]

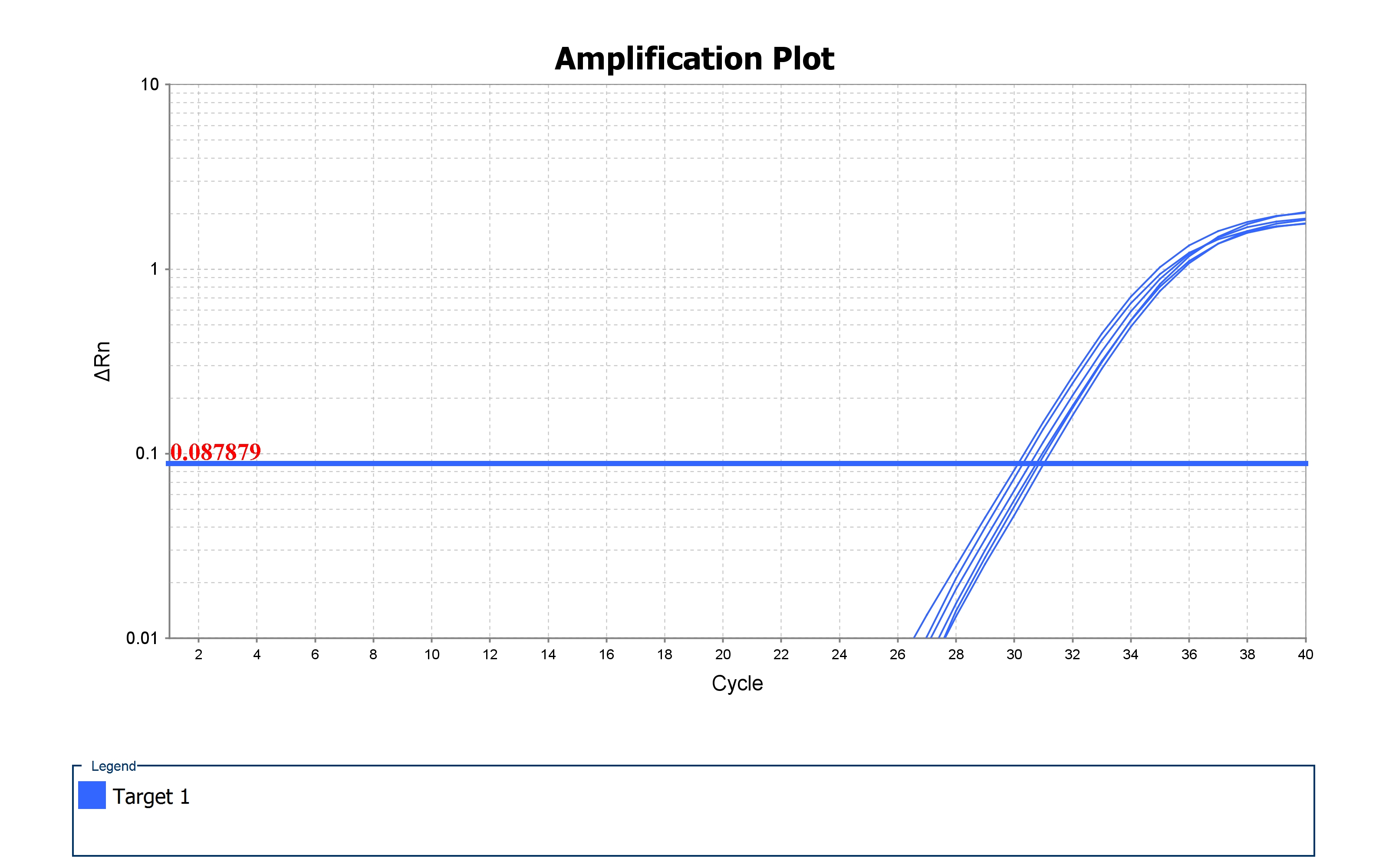

Supplement: Supplementary file 2 — Supplementary Material 2. [file 12864_2025_12244_MOESM2_ESM.zip › Supplementary file2-Amplification Plot/poly(A) tailing/aly-miR159c-3p.jpg]

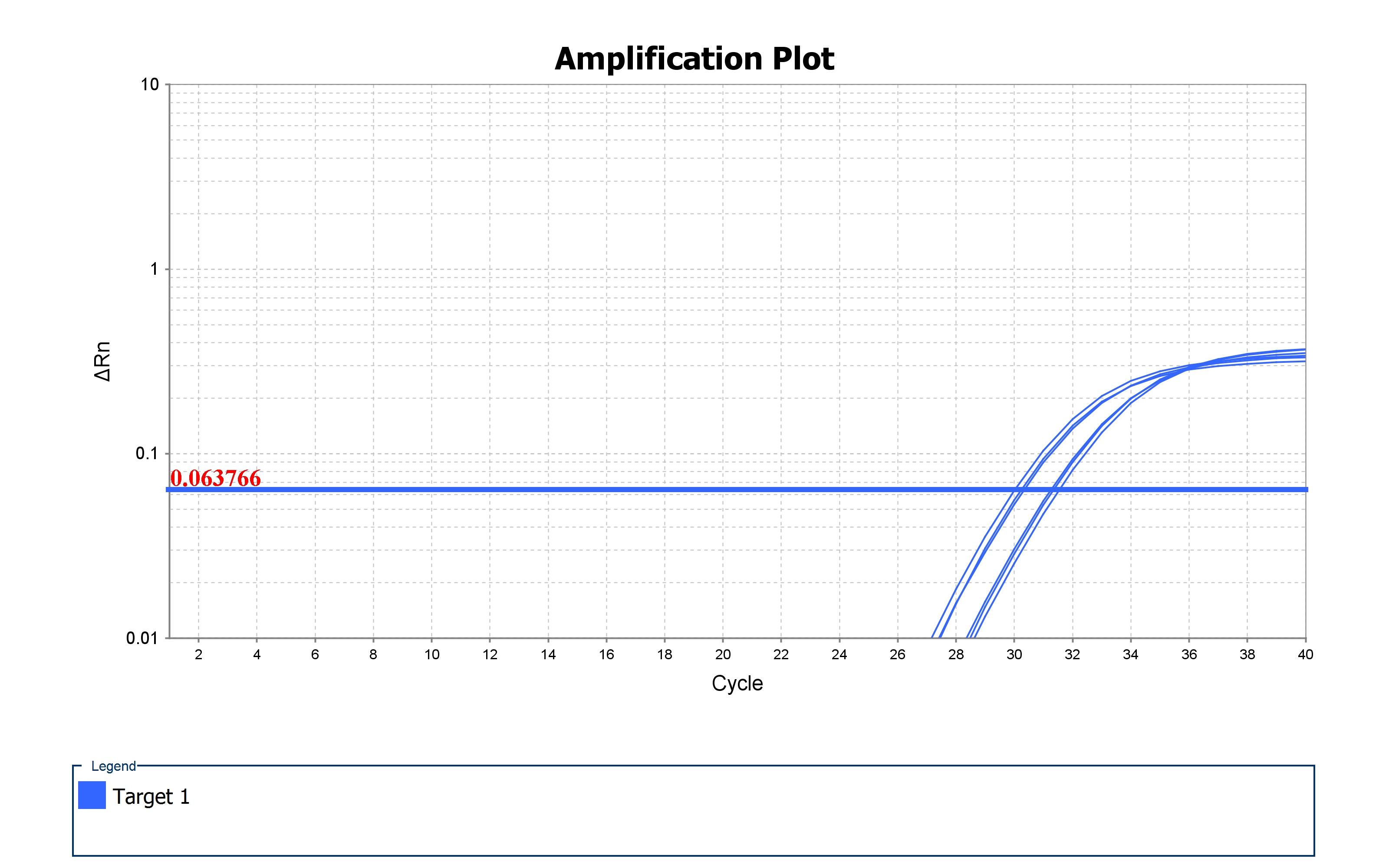

Supplement: Supplementary file 2 — Supplementary Material 2. [file 12864_2025_12244_MOESM2_ESM.zip › Supplementary file2-Amplification Plot/poly(A) tailing/aly-miR166a-5p.jpg]

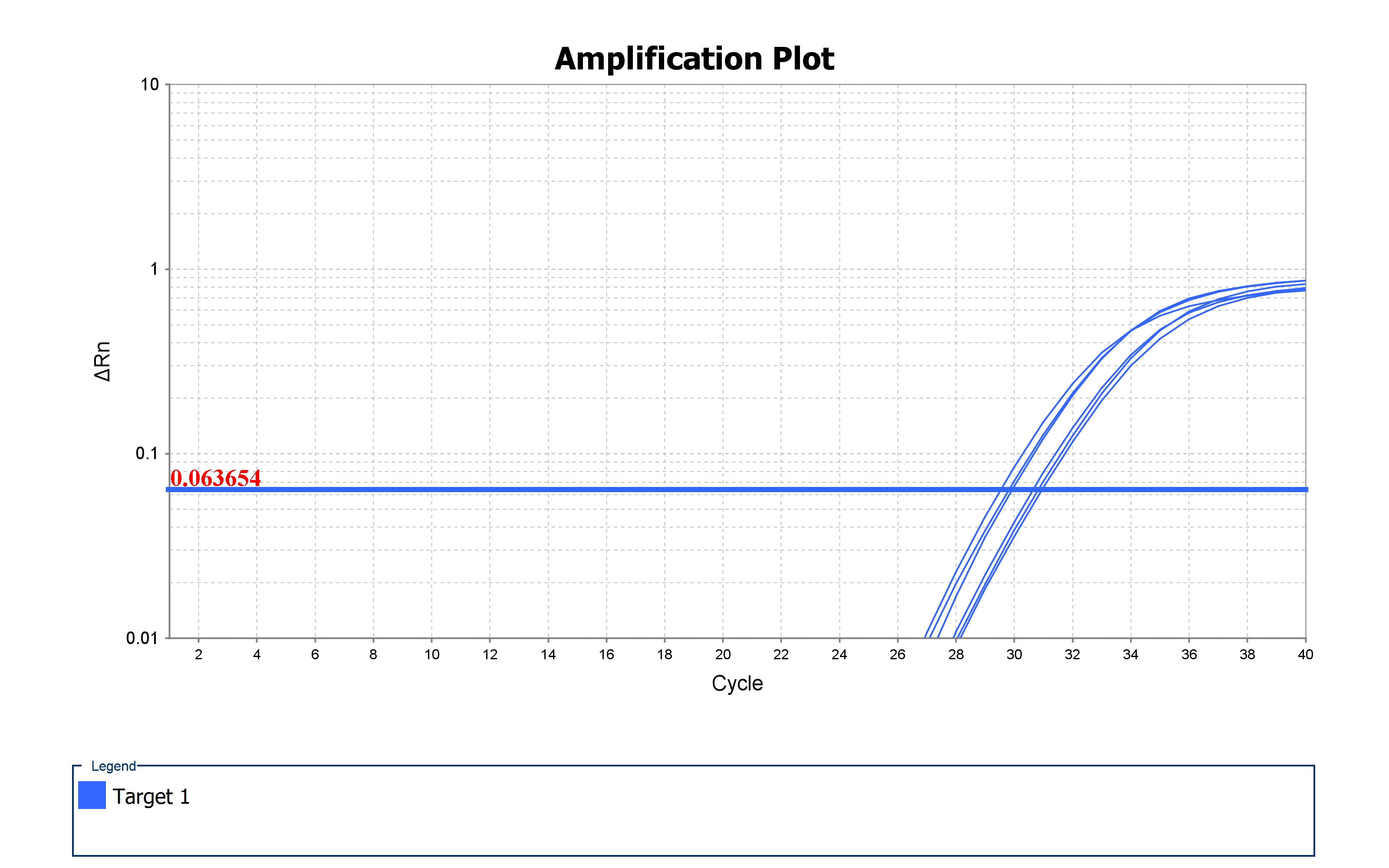

Supplement: Supplementary file 2 — Supplementary Material 2. [file 12864_2025_12244_MOESM2_ESM.zip › Supplementary file2-Amplification Plot/poly(A) tailing/aly-miR396a-3p.jpg]

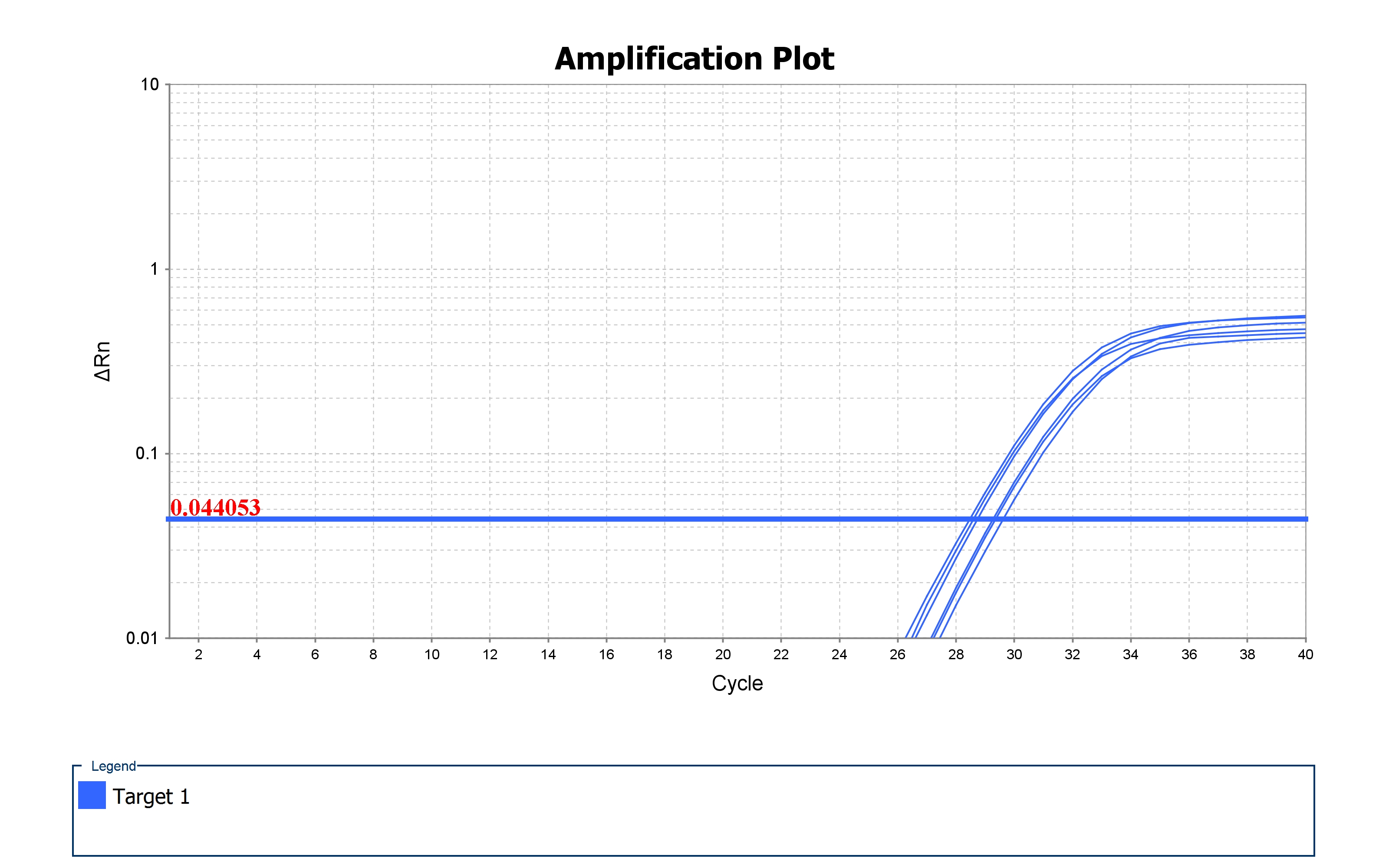

Supplement: Supplementary file 2 — Supplementary Material 2. [file 12864_2025_12244_MOESM2_ESM.zip › Supplementary file2-Amplification Plot/poly(A) tailing/aly-miR396b-5p.jpg]

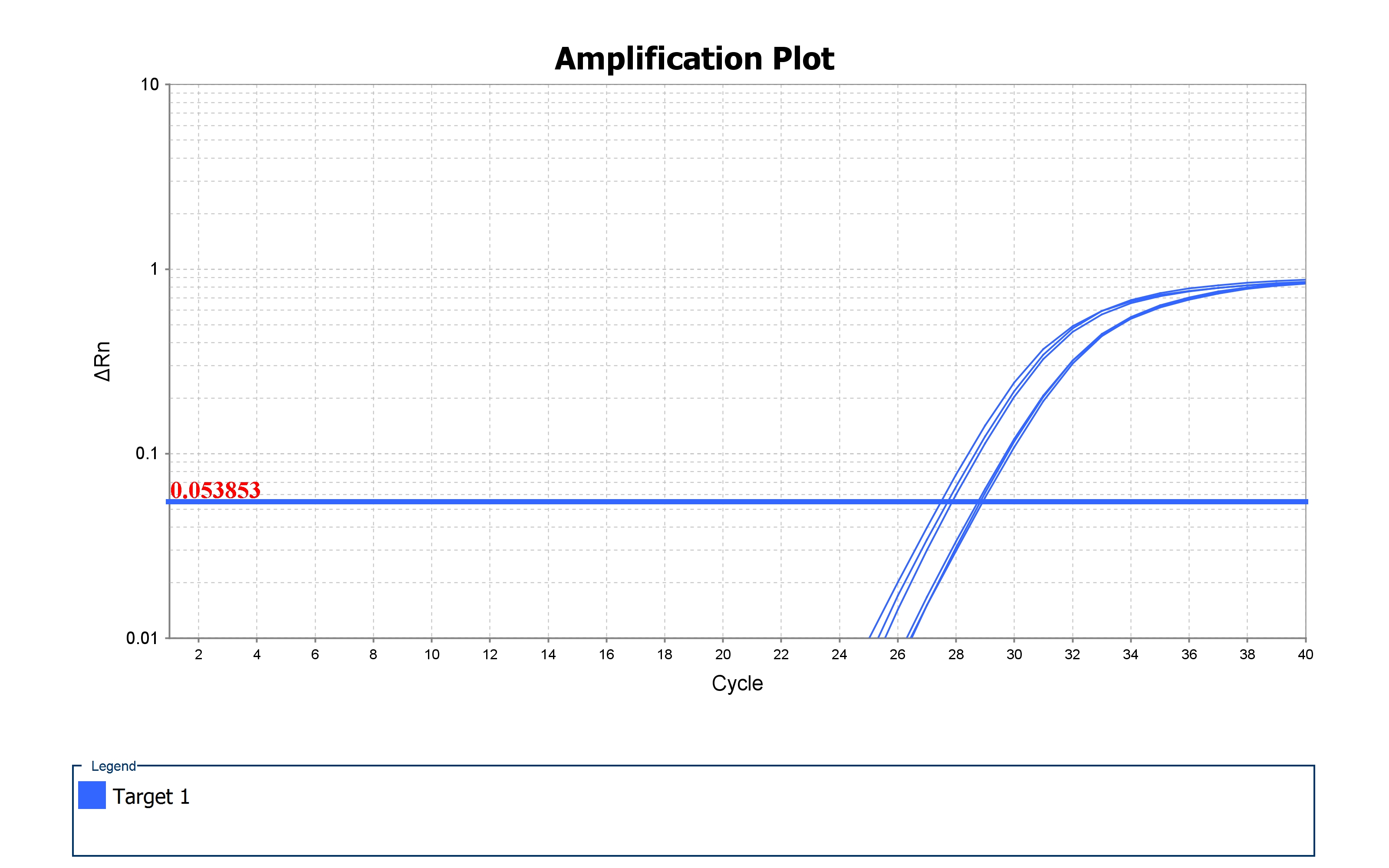

Supplement: Supplementary file 2 — Supplementary Material 2. [file 12864_2025_12244_MOESM2_ESM.zip › Supplementary file2-Amplification Plot/poly(A) tailing/aof-miR166a.jpg]

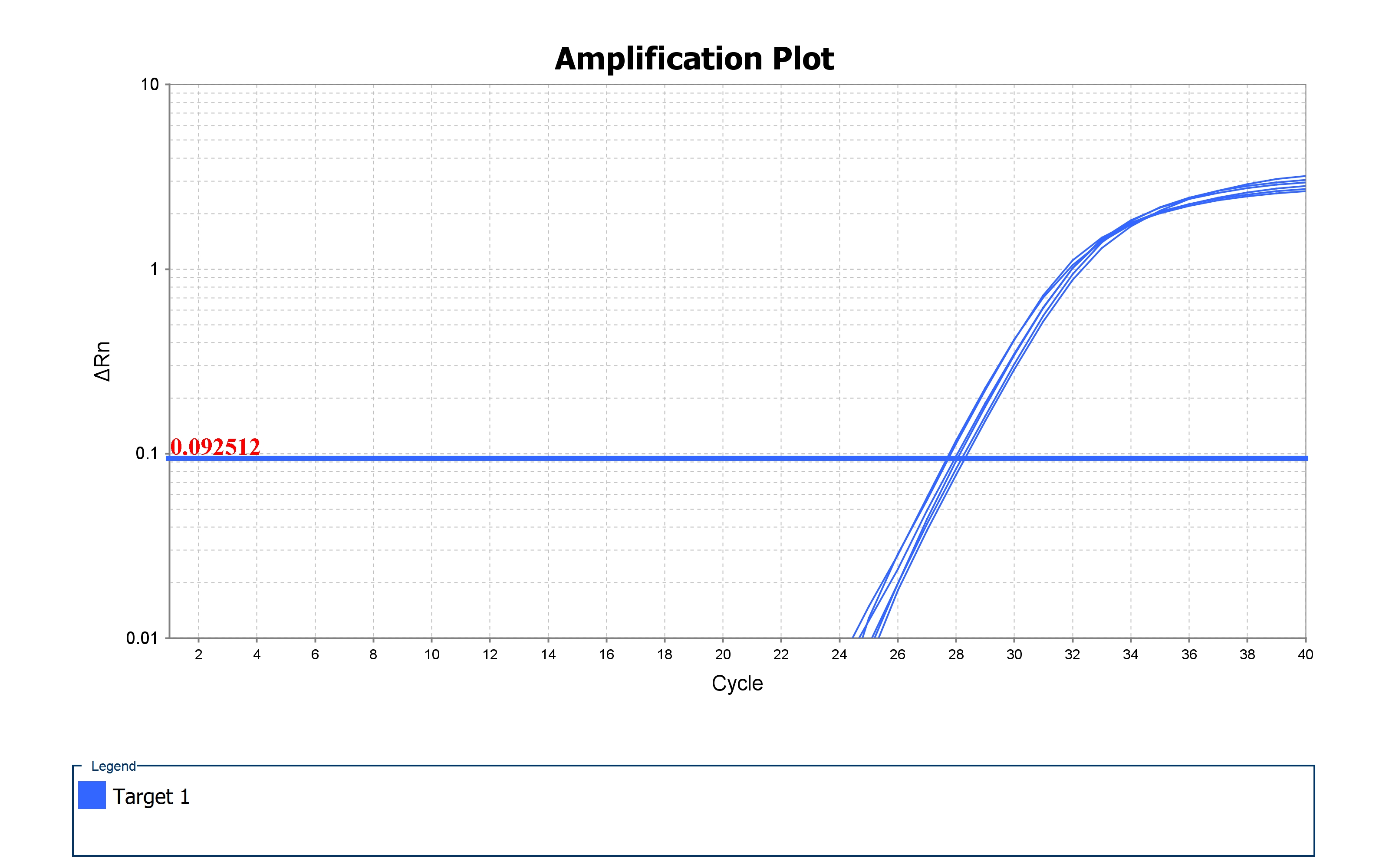

Supplement: Supplementary file 2 — Supplementary Material 2. [file 12864_2025_12244_MOESM2_ESM.zip › Supplementary file2-Amplification Plot/poly(A) tailing/aof-miR398.jpg]

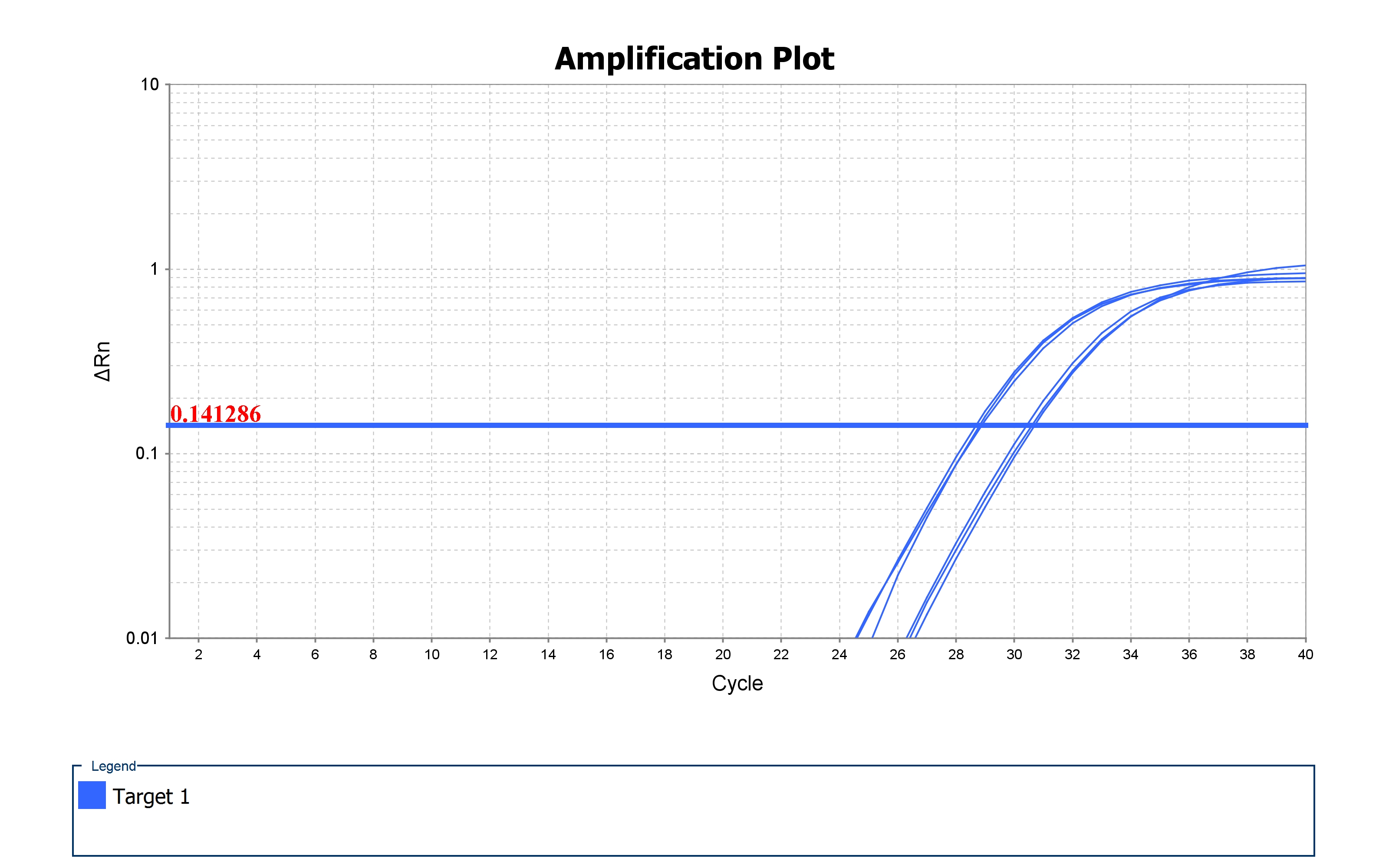

Supplement: Supplementary file 2 — Supplementary Material 2. [file 12864_2025_12244_MOESM2_ESM.zip › Supplementary file2-Amplification Plot/poly(A) tailing/ata-miR396a-5p.jpg]

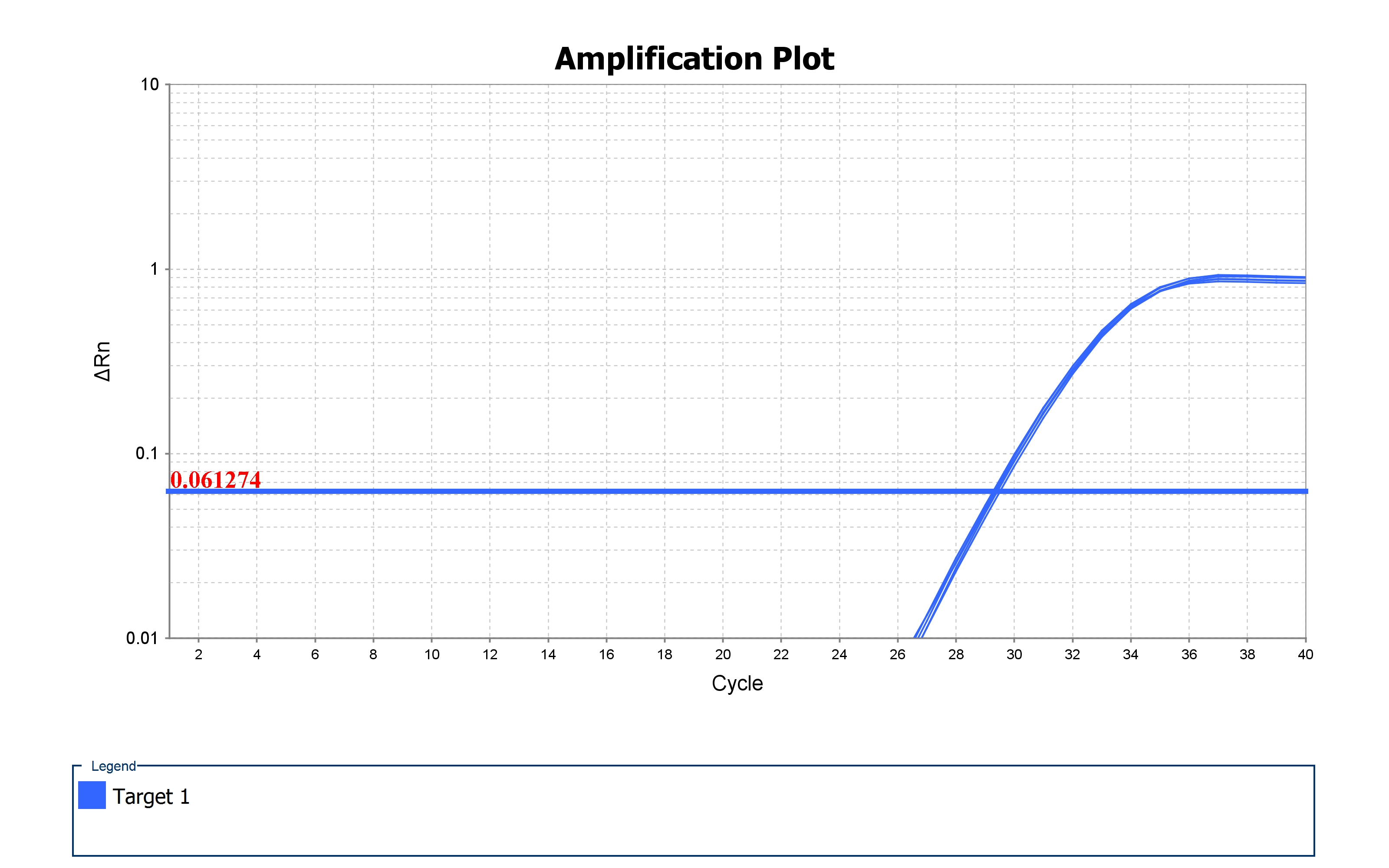

Supplement: Supplementary file 2 — Supplementary Material 2. [file 12864_2025_12244_MOESM2_ESM.zip › Supplementary file2-Amplification Plot/poly(A) tailing/bdi-miR159b-3p.1.jpg]

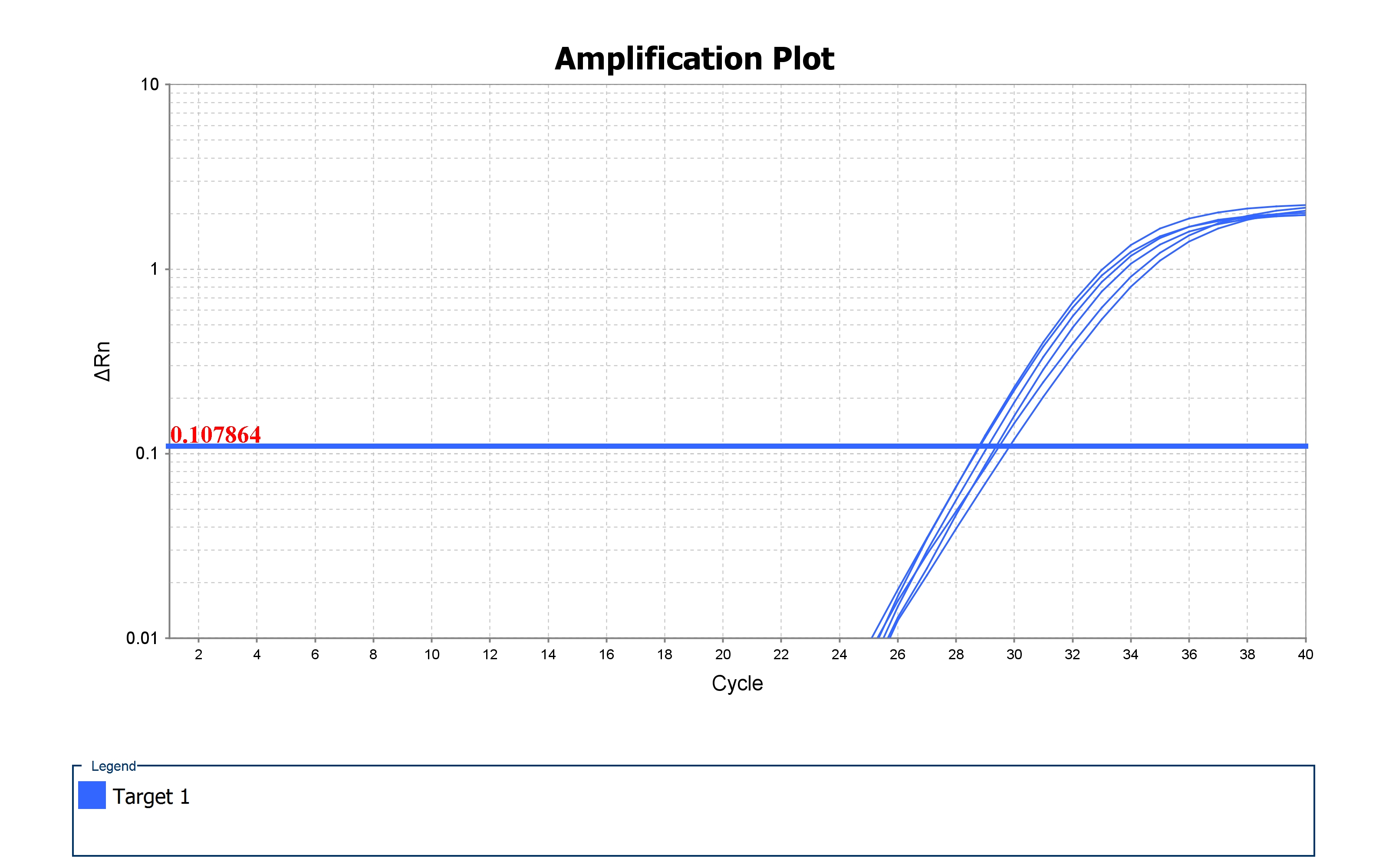

Supplement: Supplementary file 2 — Supplementary Material 2. [file 12864_2025_12244_MOESM2_ESM.zip › Supplementary file2-Amplification Plot/poly(A) tailing/bdi-miR162.jpg]

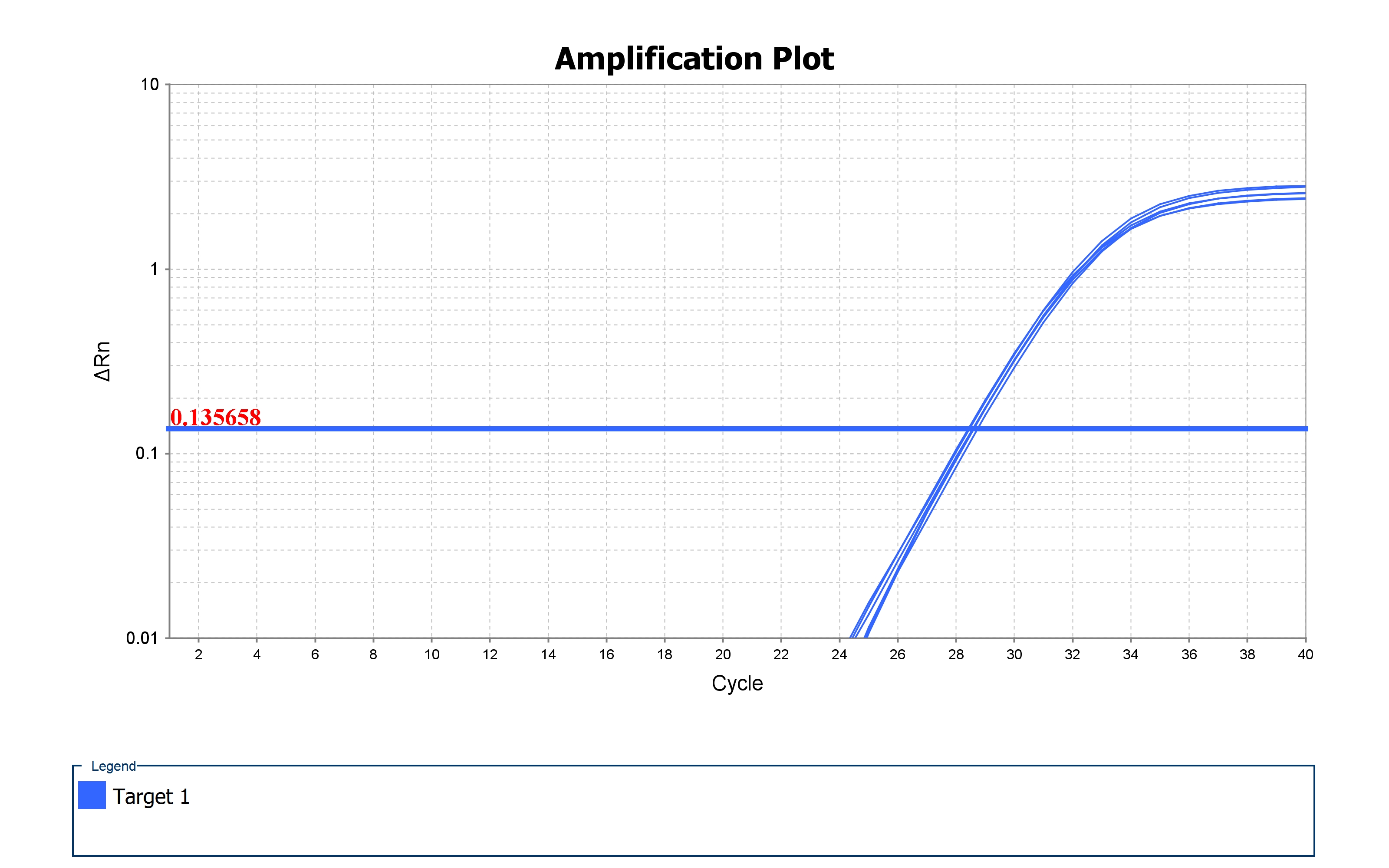

Supplement: Supplementary file 2 — Supplementary Material 2. [file 12864_2025_12244_MOESM2_ESM.zip › Supplementary file2-Amplification Plot/poly(A) tailing/bdi-miR319b-3p.jpg]

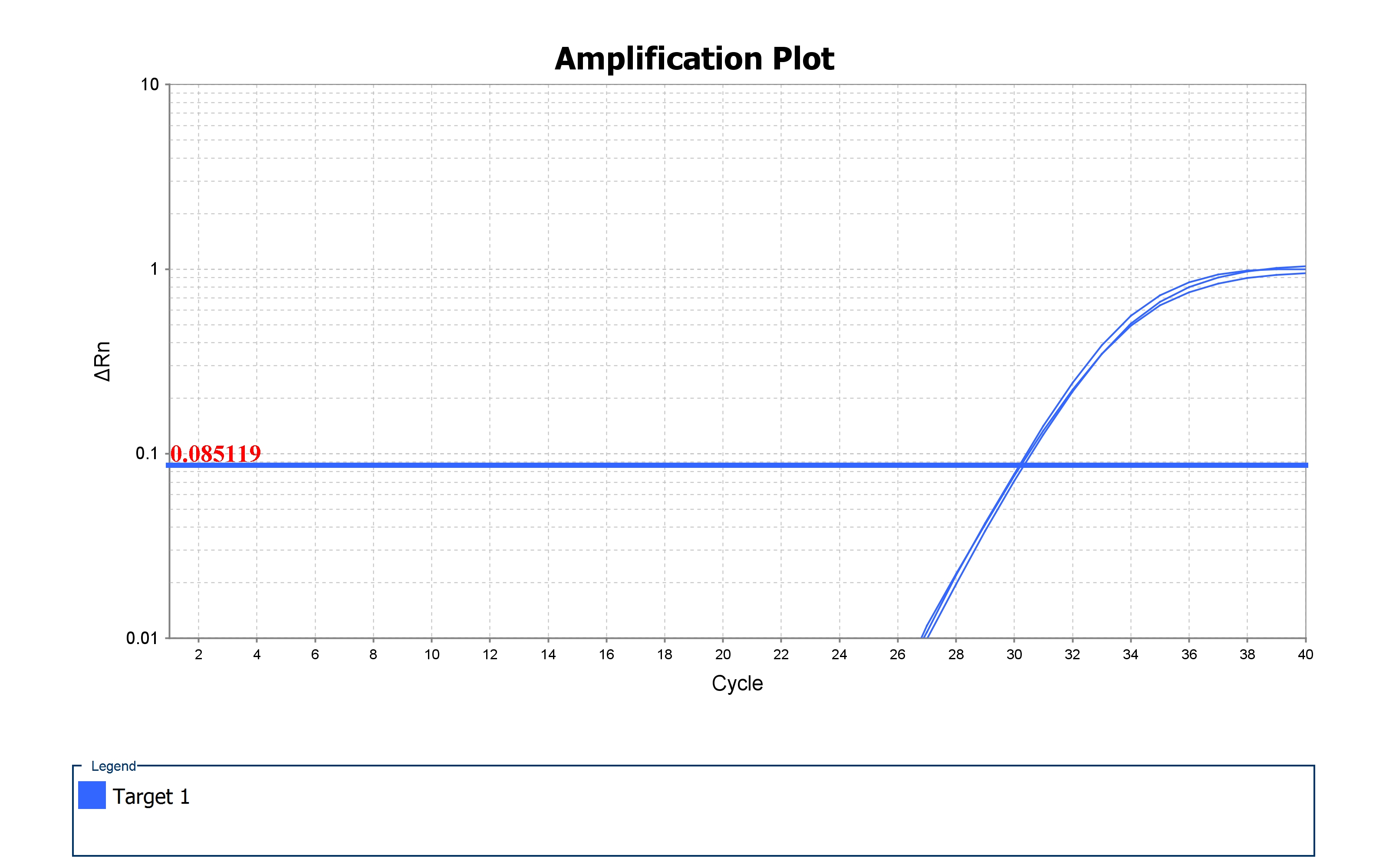

Supplement: Supplementary file 2 — Supplementary Material 2. [file 12864_2025_12244_MOESM2_ESM.zip › Supplementary file2-Amplification Plot/poly(A) tailing/cas-miR166c-3p.jpg]

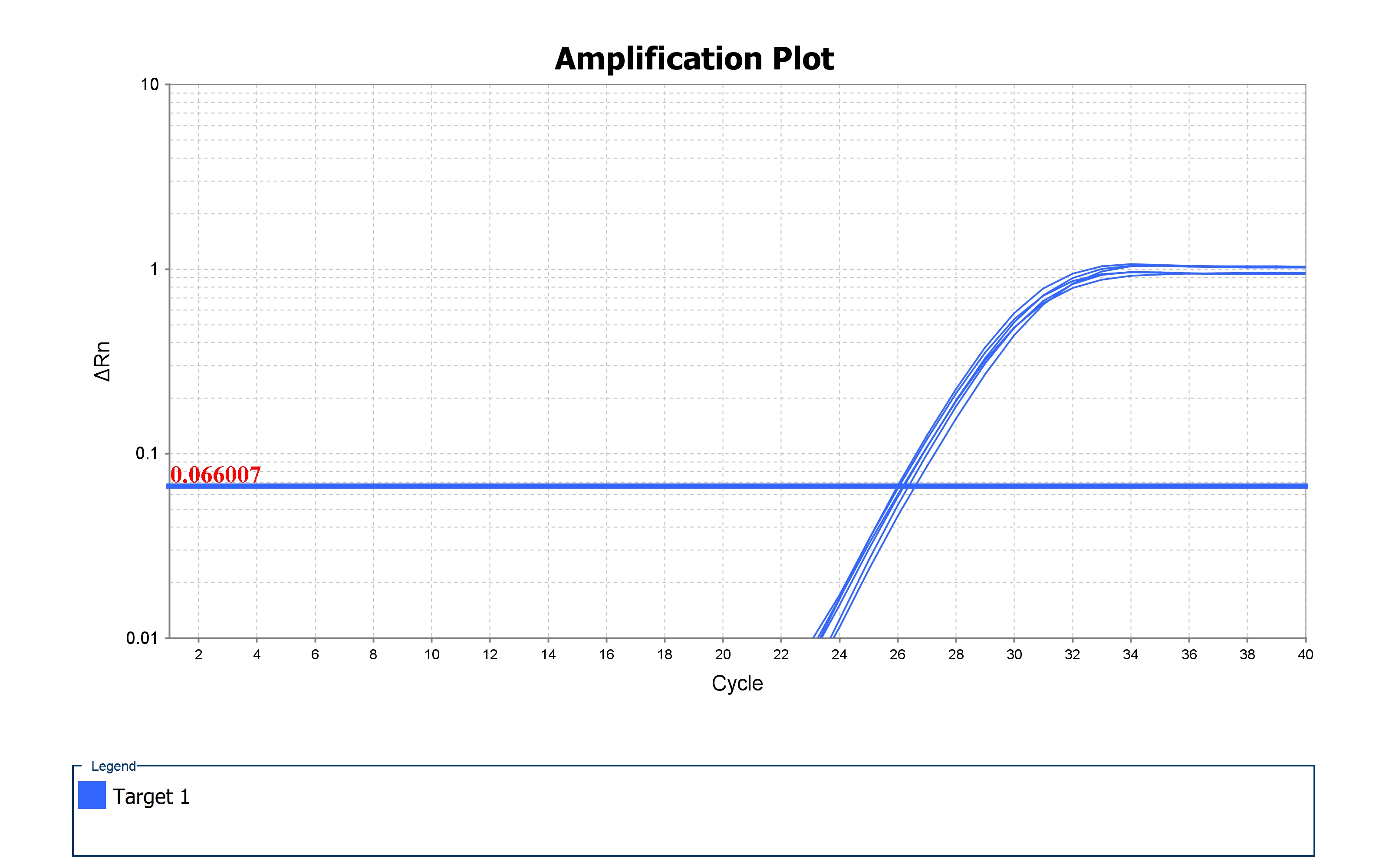

Supplement: Supplementary file 2 — Supplementary Material 2. [file 12864_2025_12244_MOESM2_ESM.zip › Supplementary file2-Amplification Plot/poly(A) tailing/fve-miR1511.jpg]

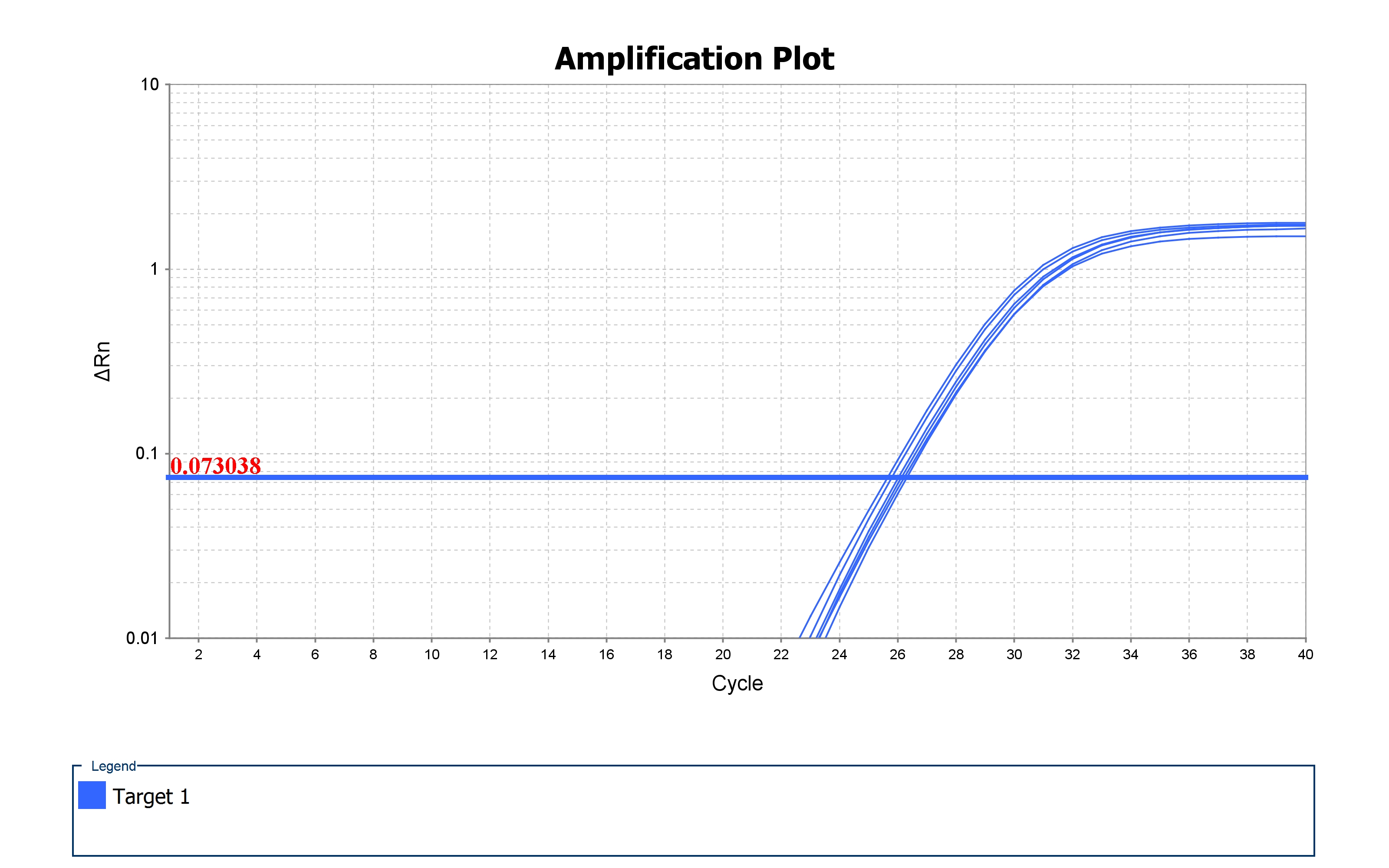

Supplement: Supplementary file 2 — Supplementary Material 2. [file 12864_2025_12244_MOESM2_ESM.zip › Supplementary file2-Amplification Plot/poly(A) tailing/hbr-miR6173.jpg]

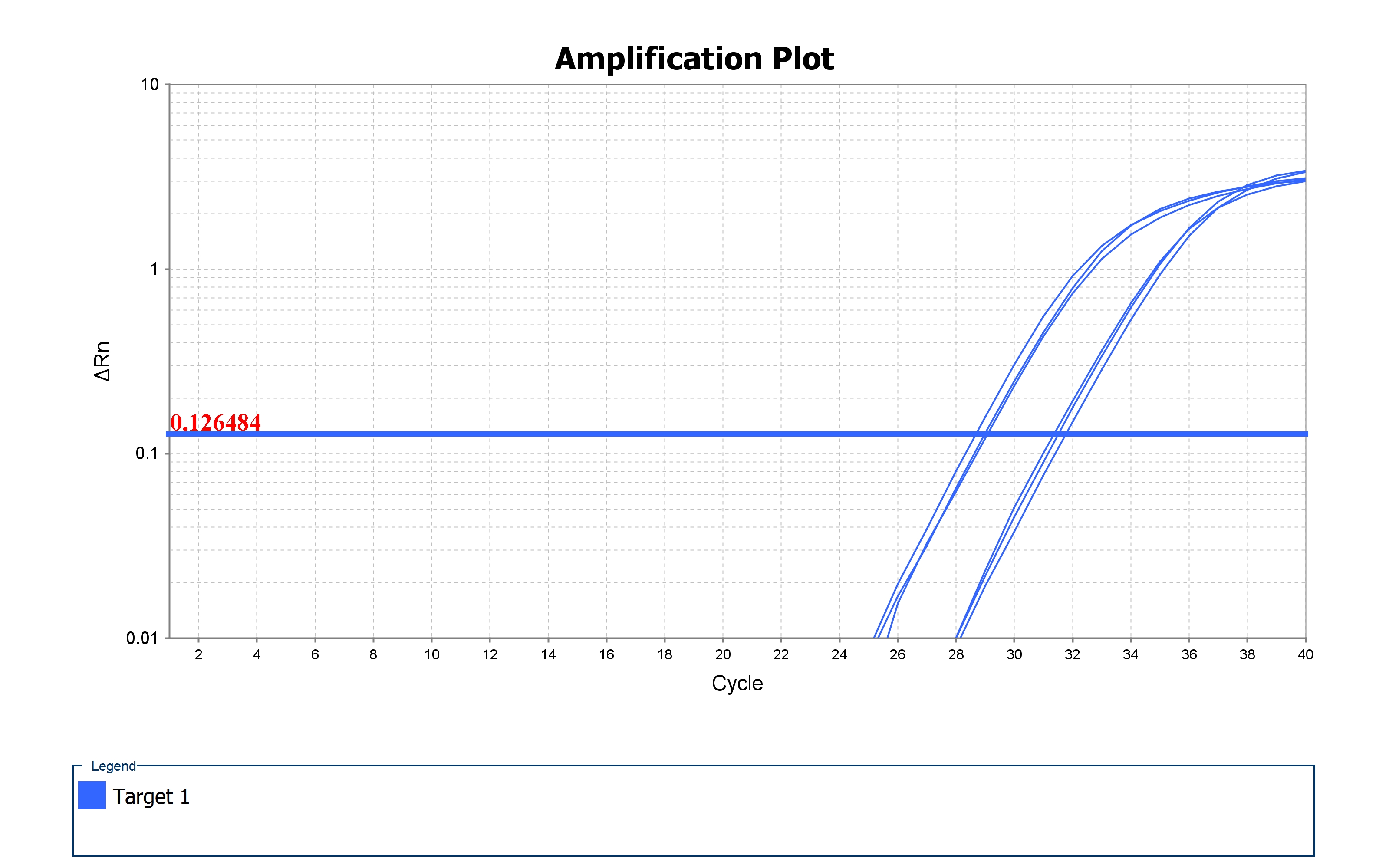

Supplement: Supplementary file 2 — Supplementary Material 2. [file 12864_2025_12244_MOESM2_ESM.zip › Supplementary file2-Amplification Plot/poly(A) tailing/lja-miR166-3p.jpg]

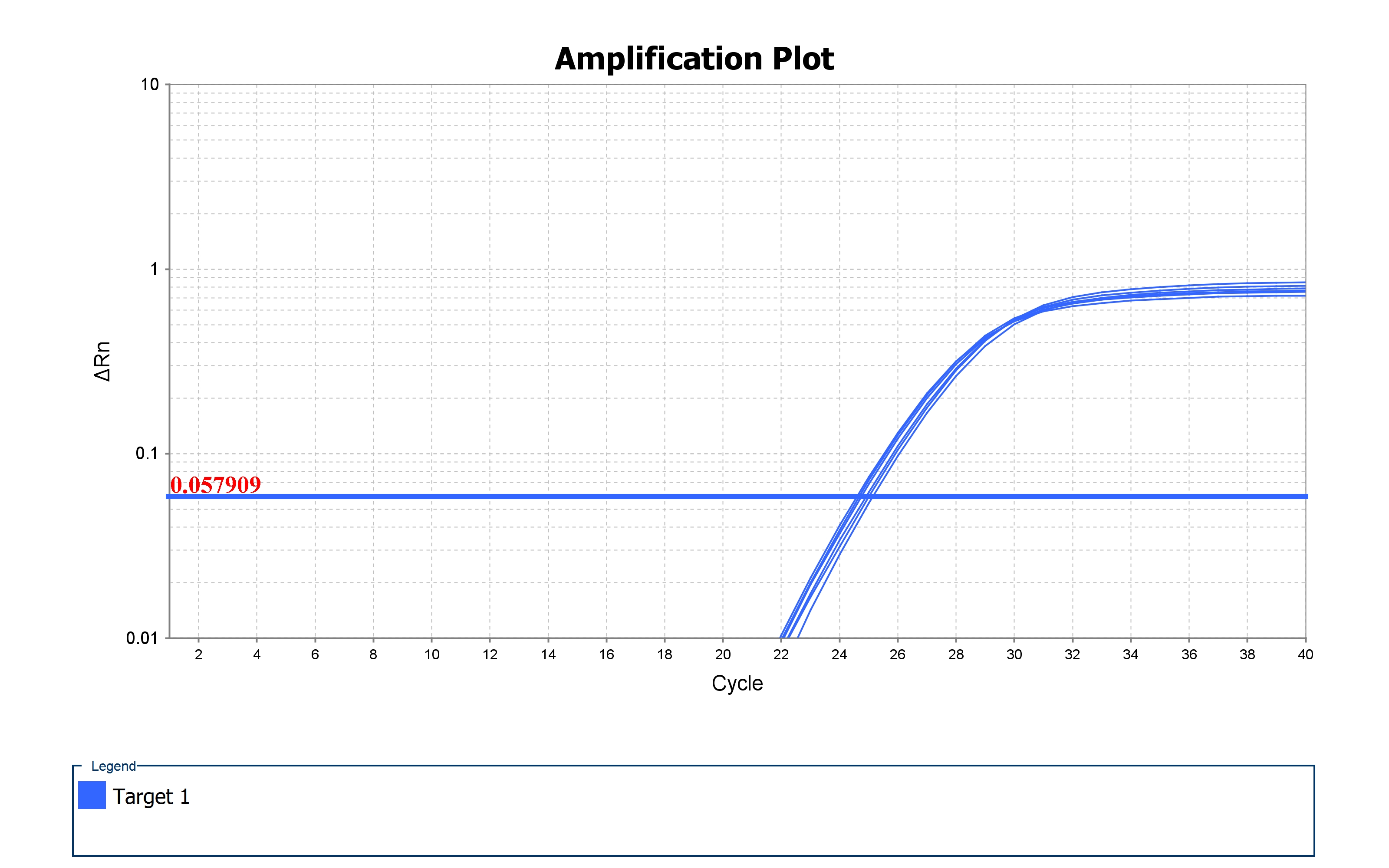

Supplement: Supplementary file 2 — Supplementary Material 2. [file 12864_2025_12244_MOESM2_ESM.zip › Supplementary file2-Amplification Plot/poly(A) tailing/lja-miR168-3p.jpg]

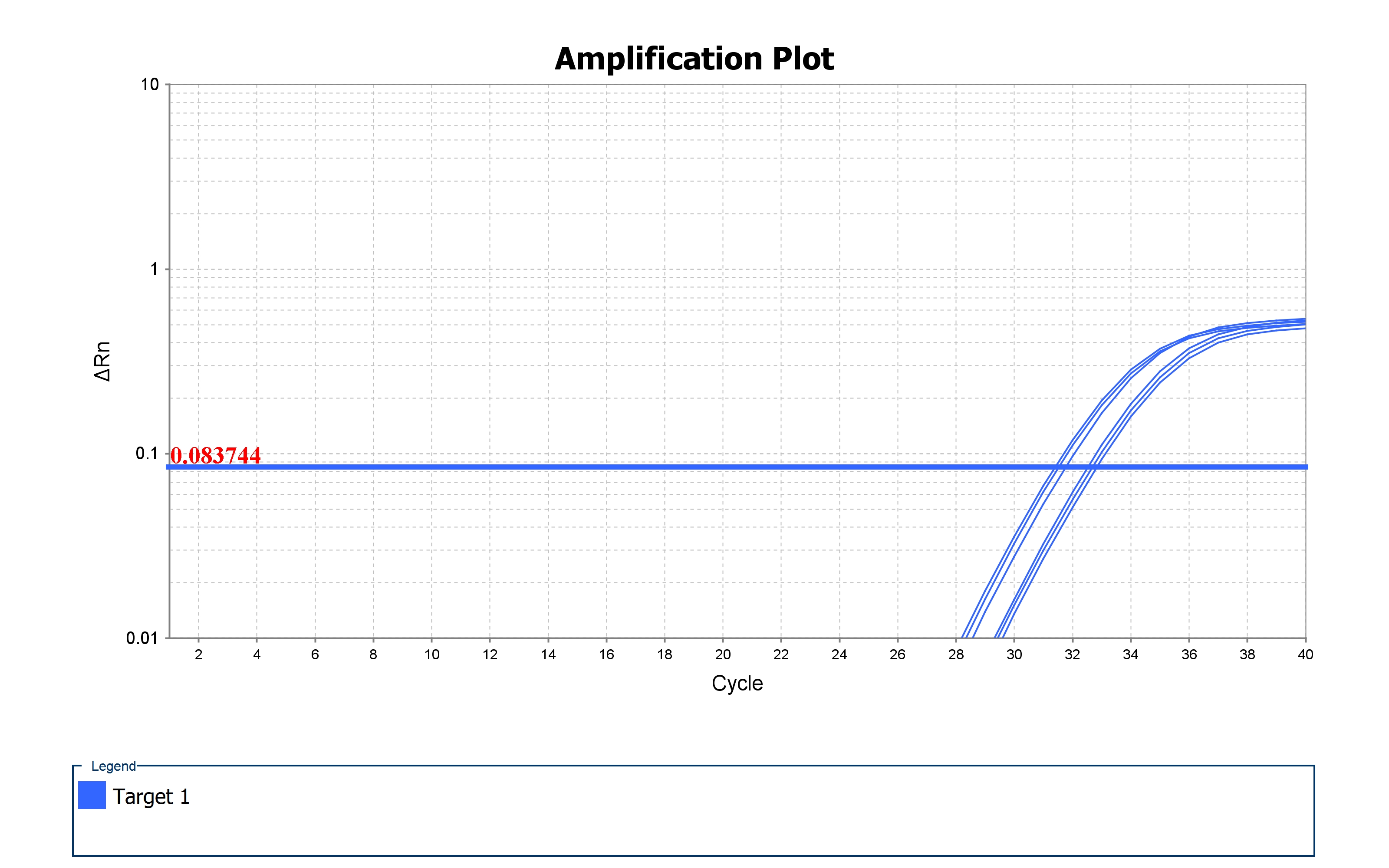

Supplement: Supplementary file 2 — Supplementary Material 2. [file 12864_2025_12244_MOESM2_ESM.zip › Supplementary file2-Amplification Plot/poly(A) tailing/pab-miR166j.jpg]

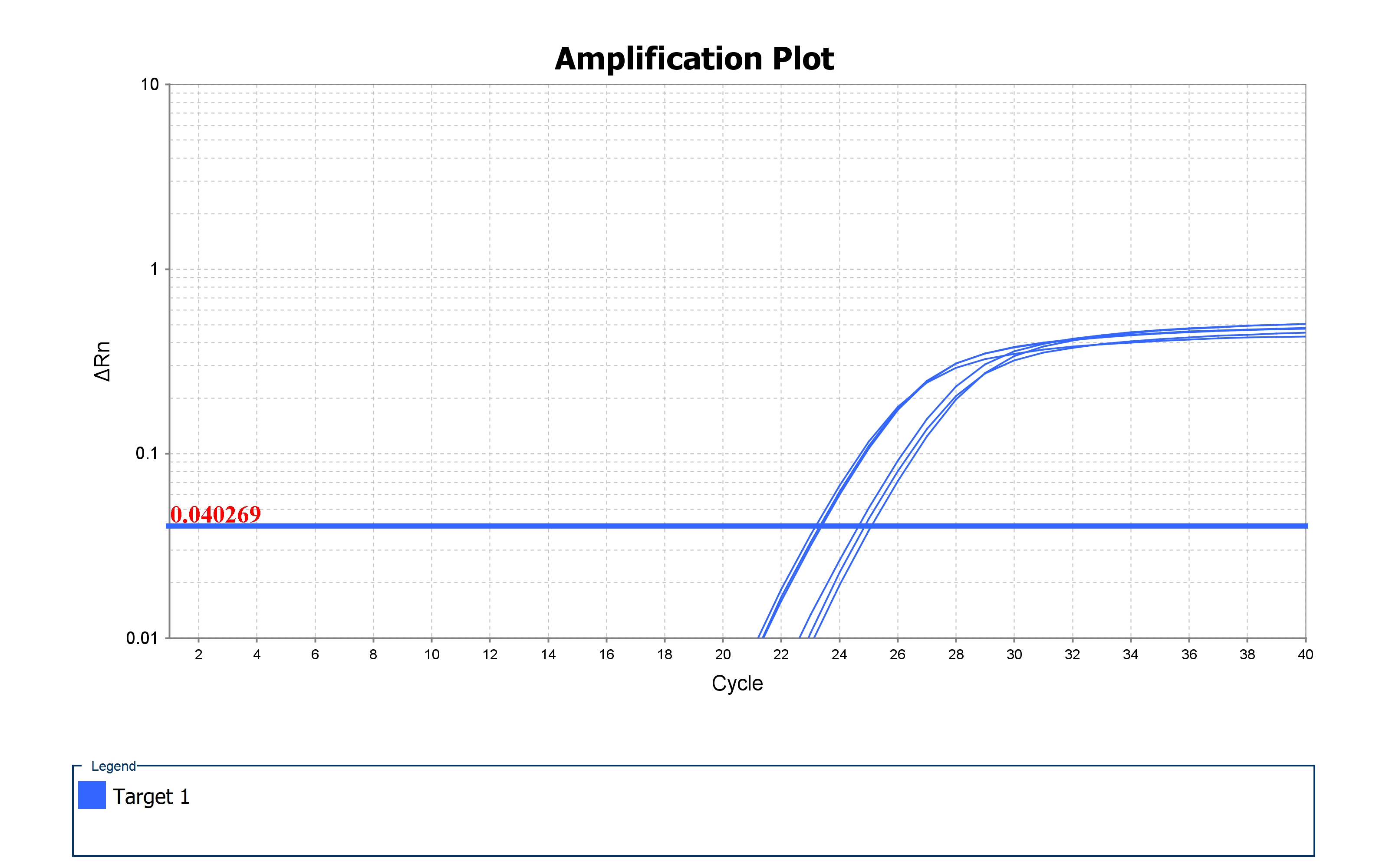

Supplement: Supplementary file 2 — Supplementary Material 2. [file 12864_2025_12244_MOESM2_ESM.zip › Supplementary file2-Amplification Plot/poly(A) tailing/peu-miR2916.jpg]

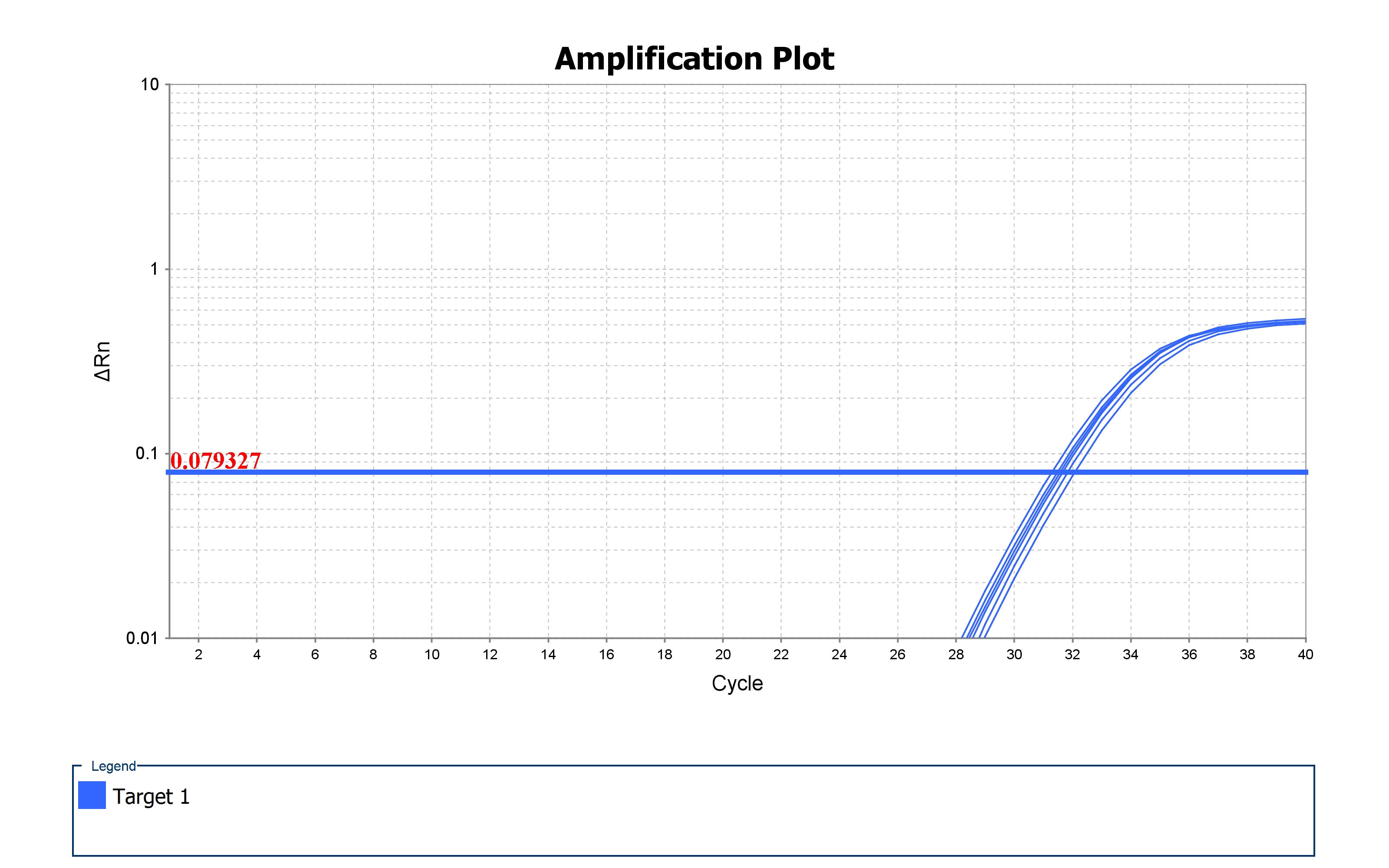

Supplement: Supplementary file 2 — Supplementary Material 2. [file 12864_2025_12244_MOESM2_ESM.zip › Supplementary file2-Amplification Plot/poly(A) tailing/pta-miR159c.jpg]

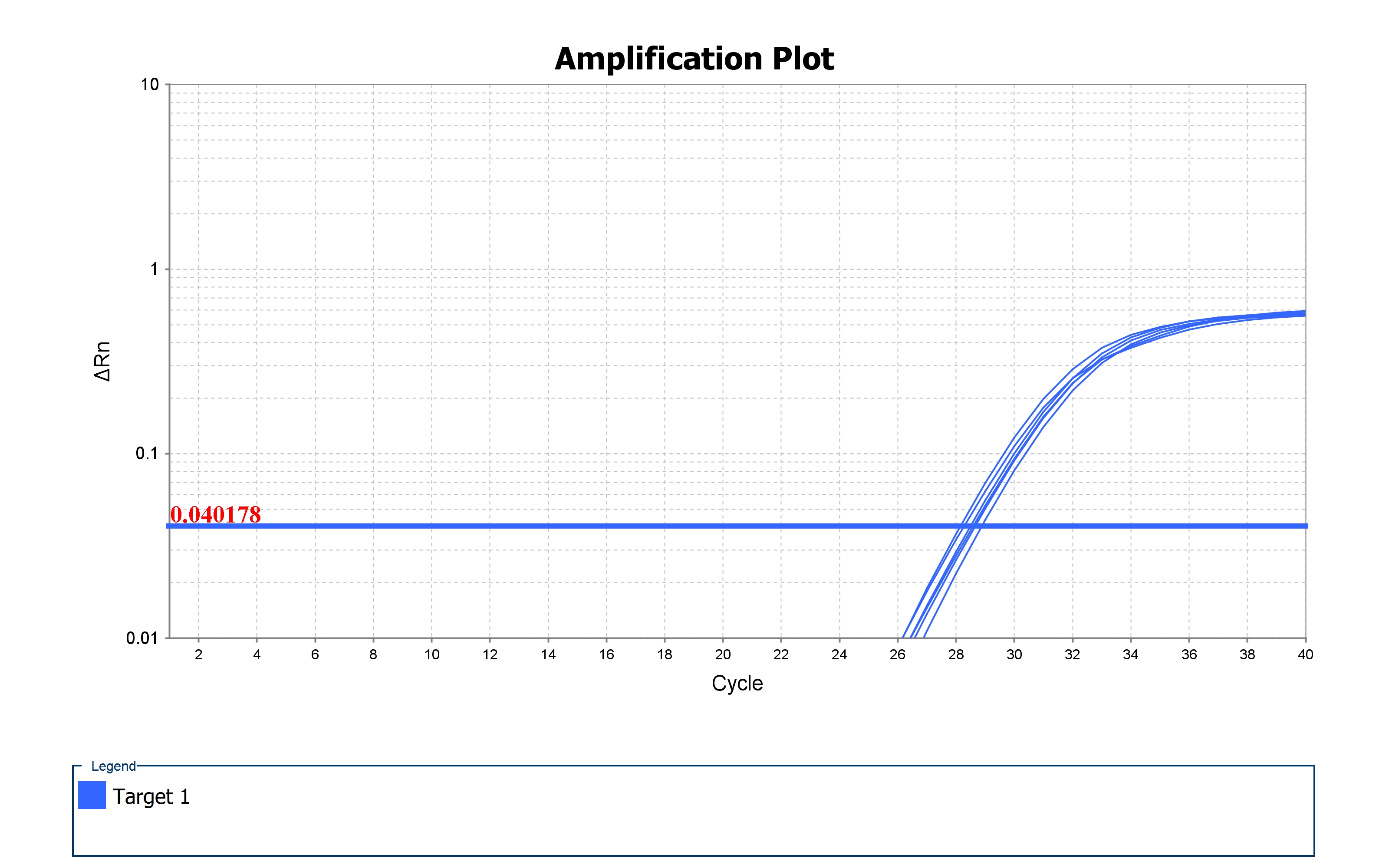

Supplement: Supplementary file 2 — Supplementary Material 2. [file 12864_2025_12244_MOESM2_ESM.zip › Supplementary file2-Amplification Plot/poly(A) tailing/ptc-miR6478.jpg]

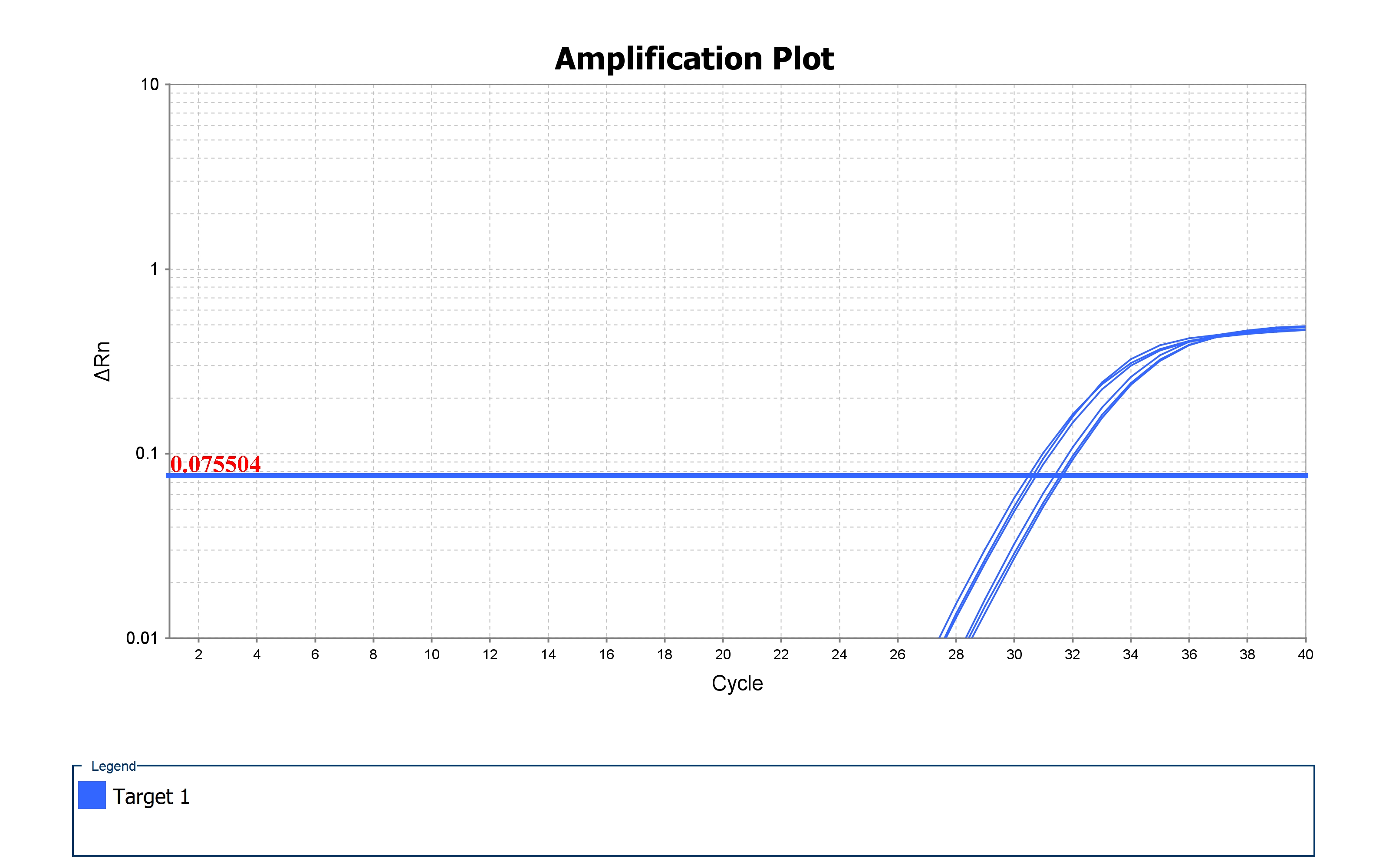

Supplement: Supplementary file 2 — Supplementary Material 2. [file 12864_2025_12244_MOESM2_ESM.zip › Supplementary file2-Amplification Plot/poly(A) tailing/sly-miR1919a.jpg]

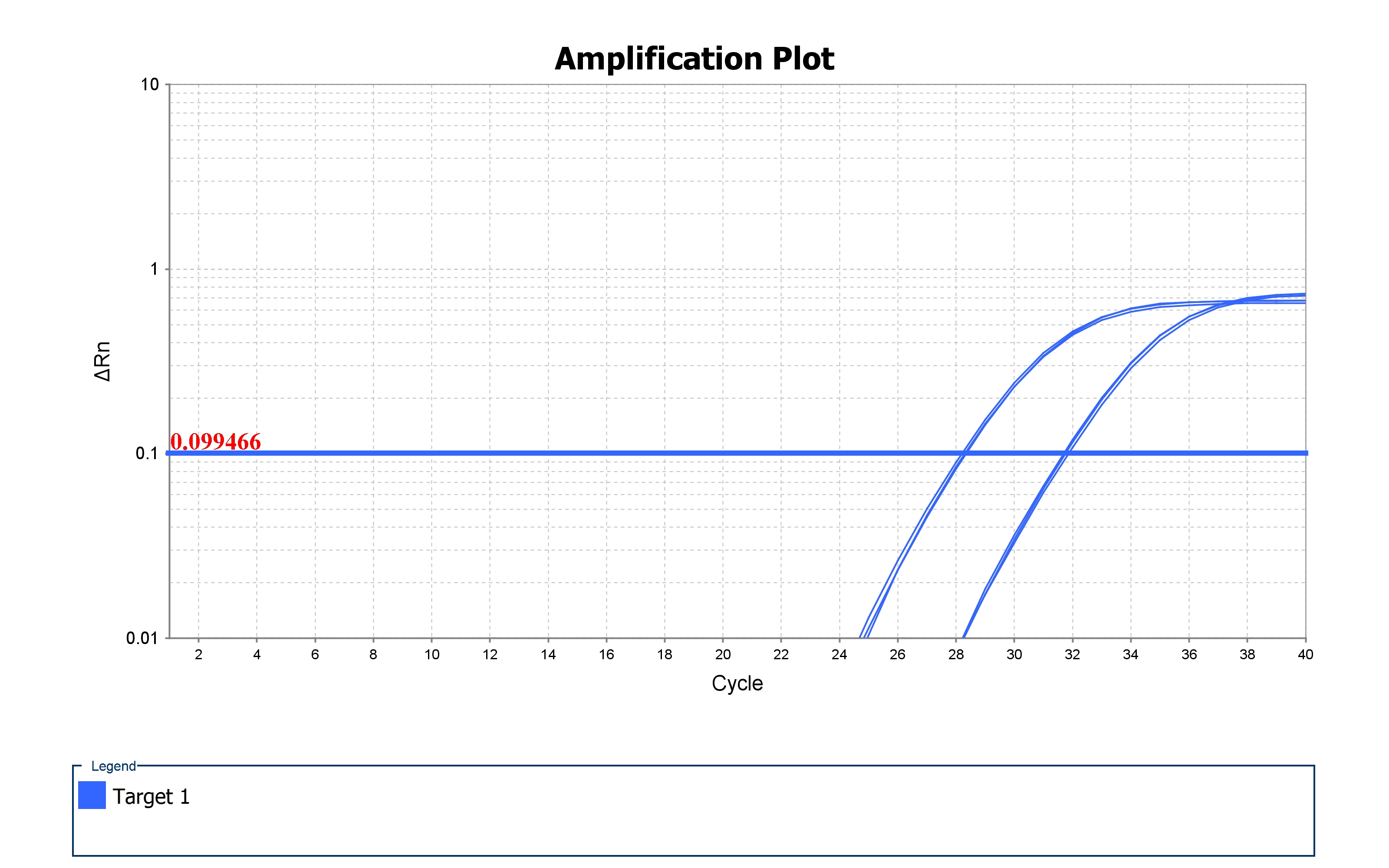

Supplement: Supplementary file 2 — Supplementary Material 2. [file 12864_2025_12244_MOESM2_ESM.zip › Supplementary file2-Amplification Plot/poly(A) tailing/sly-miR395a.jpg]

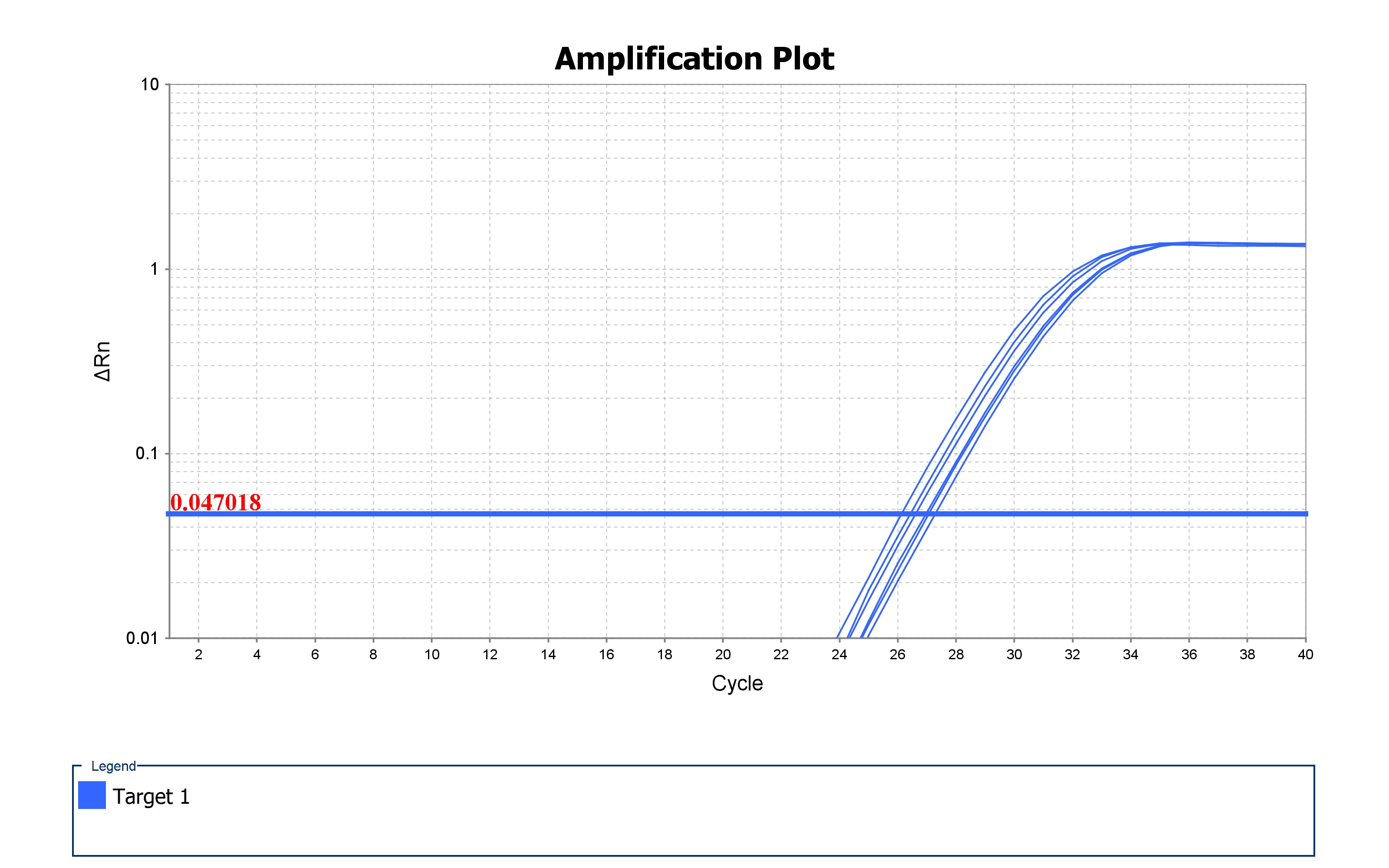

Supplement: Supplementary file 2 — Supplementary Material 2. [file 12864_2025_12244_MOESM2_ESM.zip › Supplementary file2-Amplification Plot/poly(A) tailing/sly-miR5300.jpg]

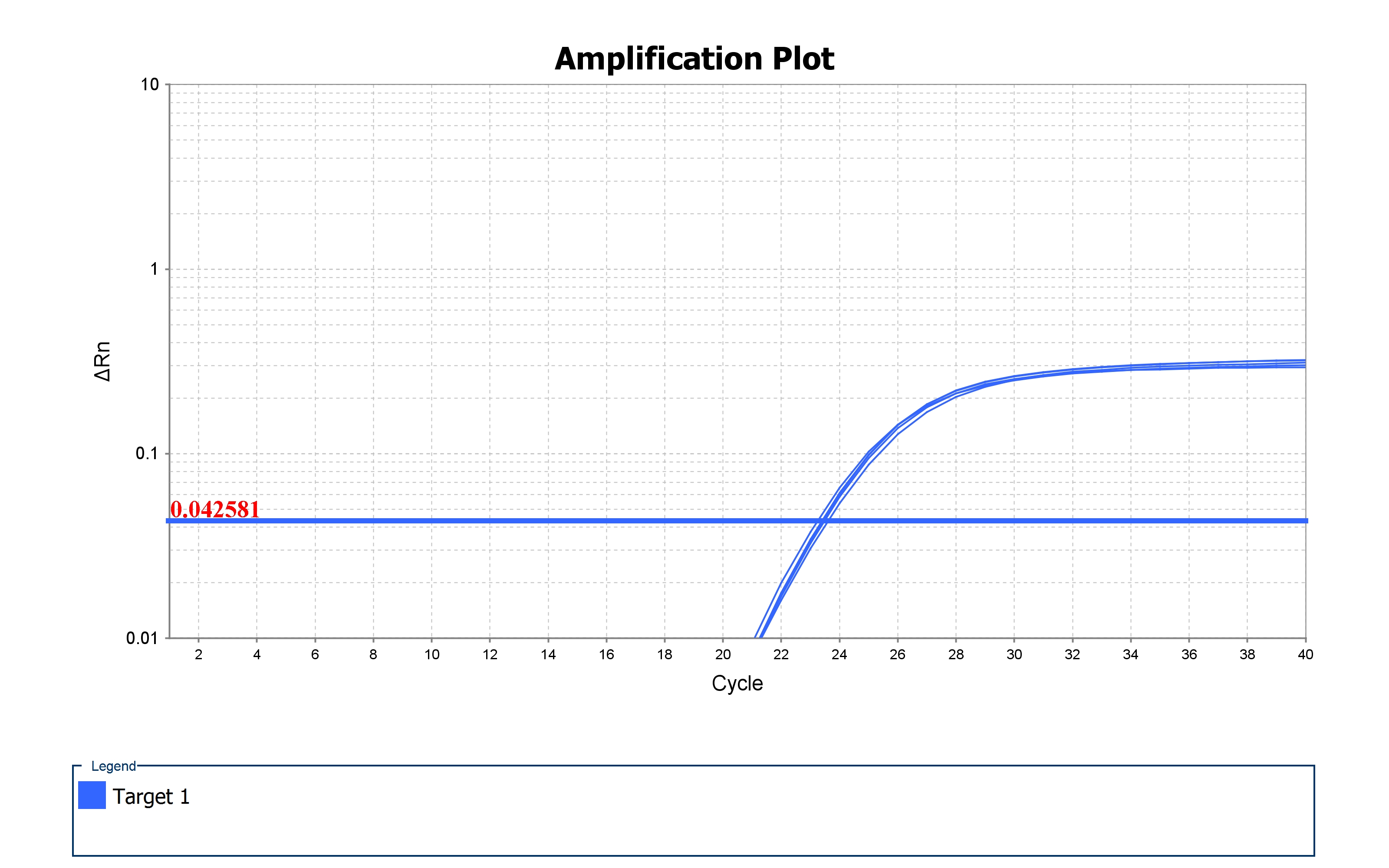

Supplement: Supplementary file 2 — Supplementary Material 2. [file 12864_2025_12244_MOESM2_ESM.zip › Supplementary file2-Amplification Plot/poly(A) tailing/U6.jpg]

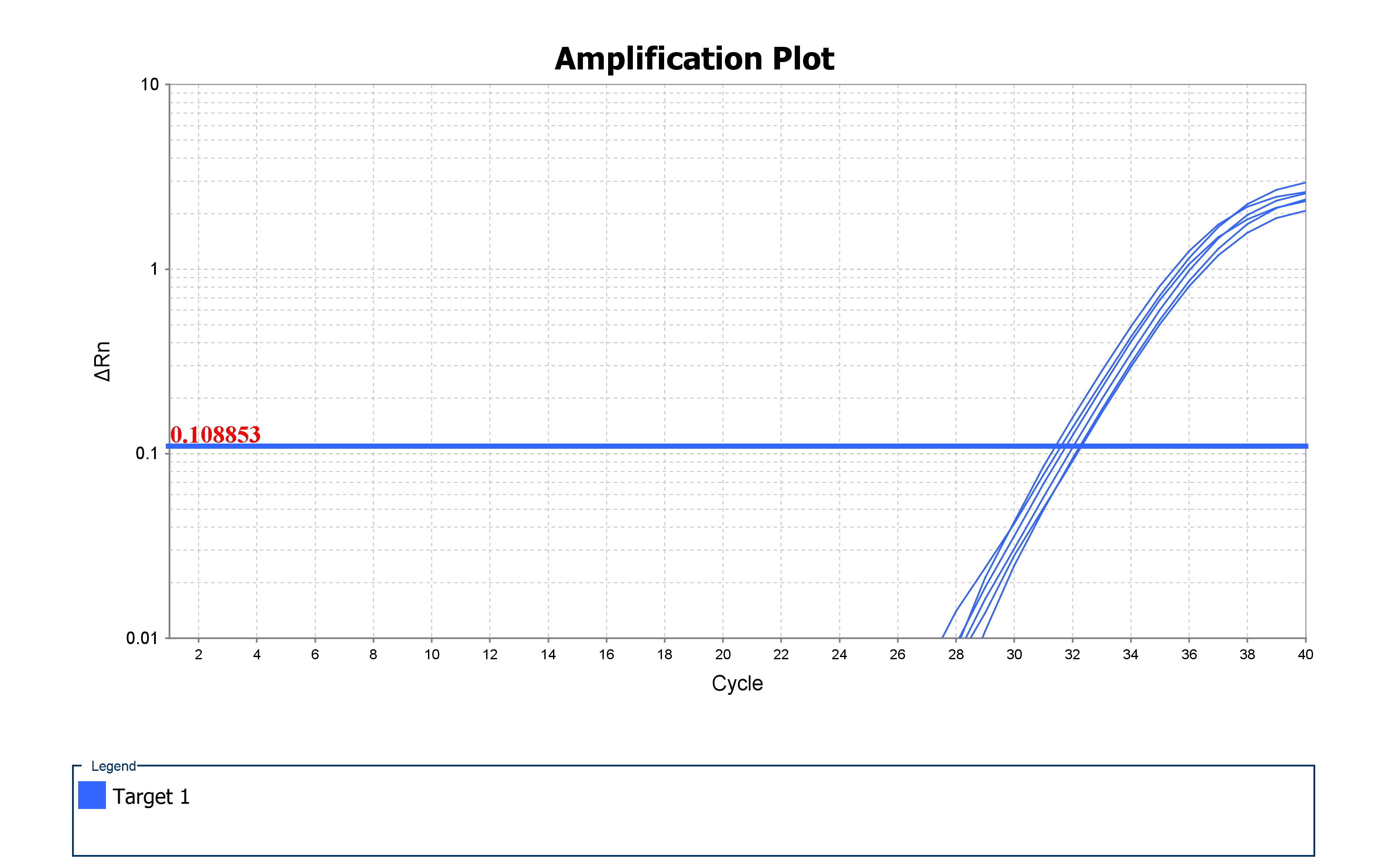

Supplement: Supplementary file 2 — Supplementary Material 2. [file 12864_2025_12244_MOESM2_ESM.zip › Supplementary file2-Amplification Plot/stem-loop/ahy-miR159.jpg]

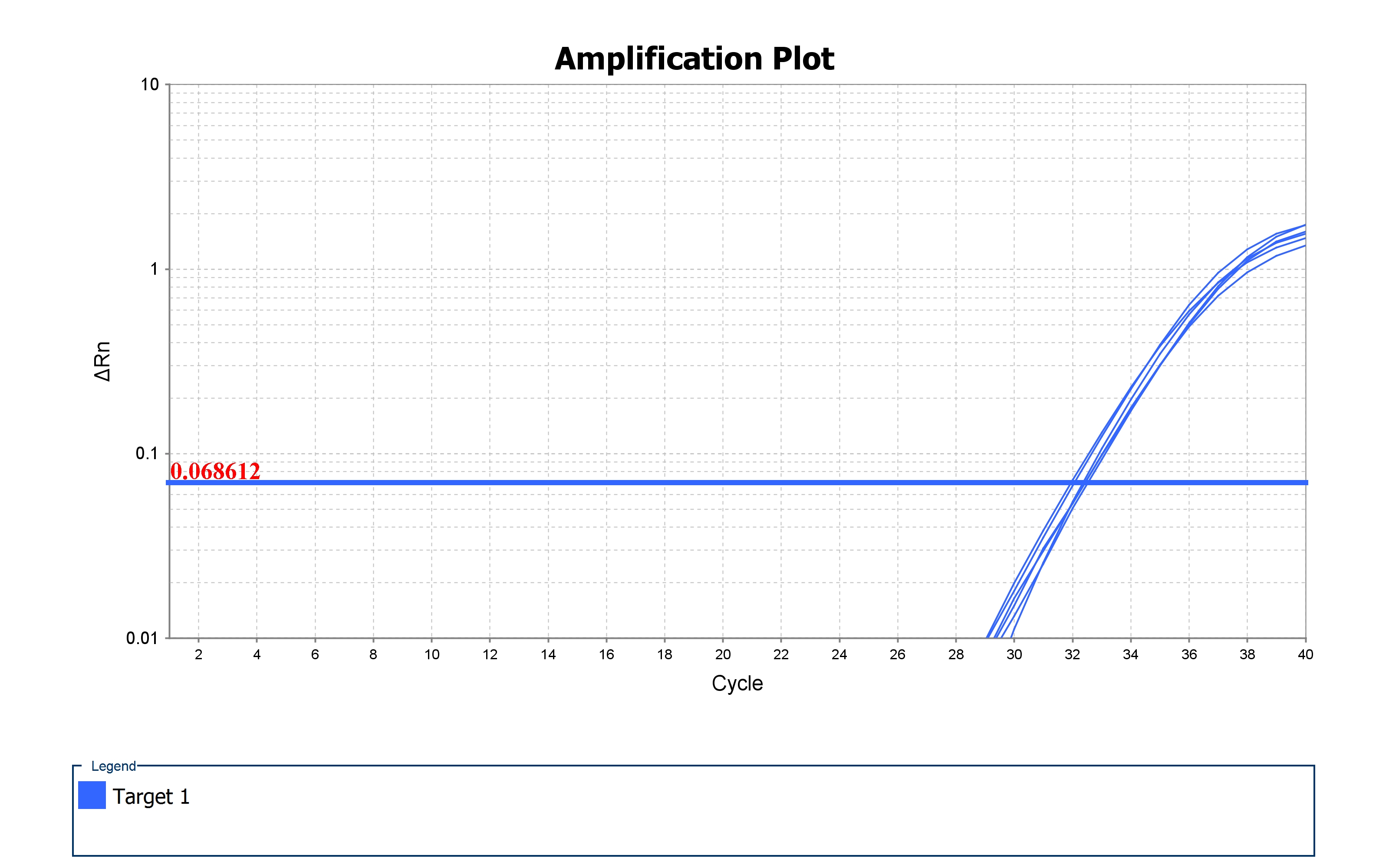

Supplement: Supplementary file 2 — Supplementary Material 2. [file 12864_2025_12244_MOESM2_ESM.zip › Supplementary file2-Amplification Plot/stem-loop/aly-miR159b-3p.jpg]

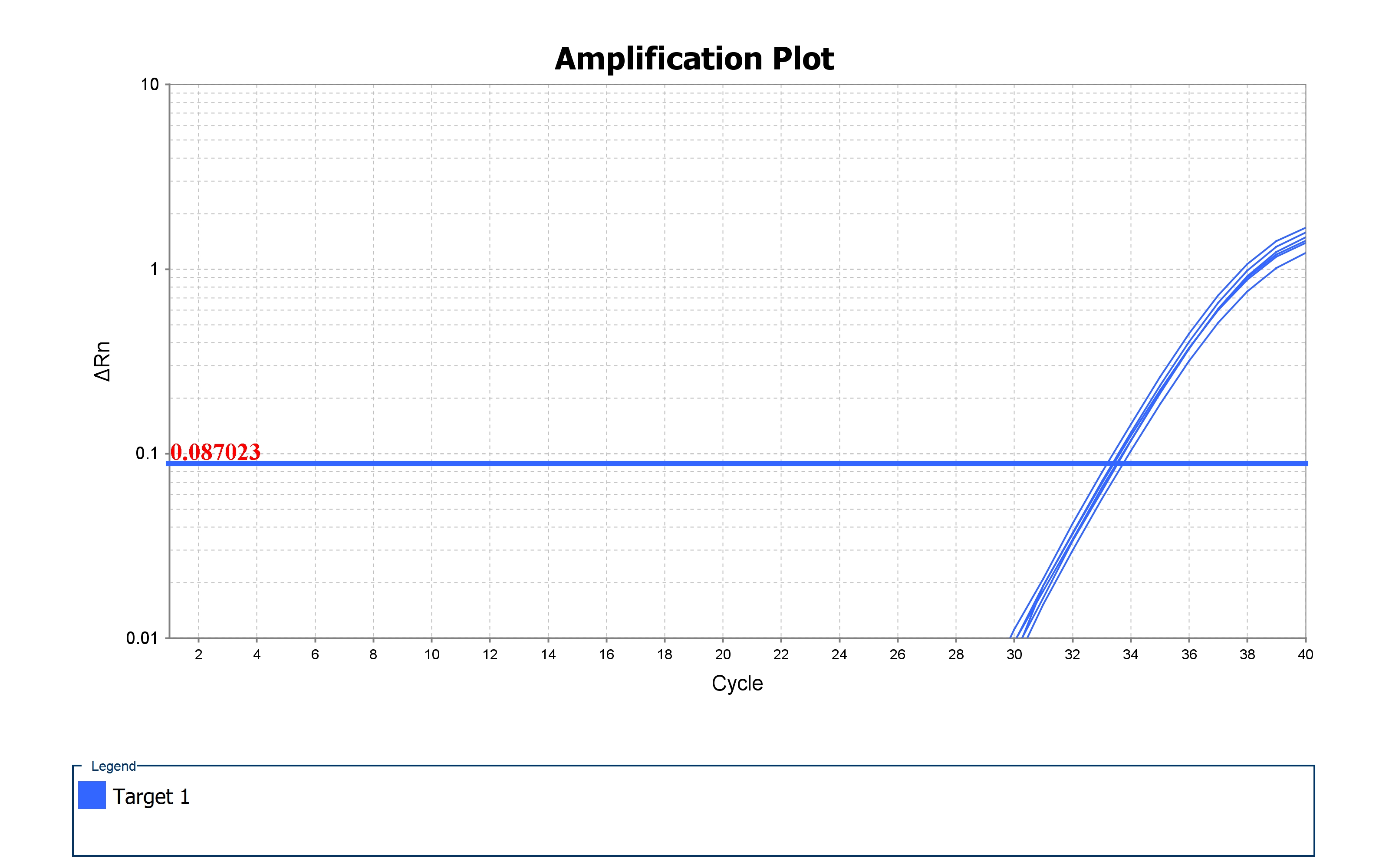

Supplement: Supplementary file 2 — Supplementary Material 2. [file 12864_2025_12244_MOESM2_ESM.zip › Supplementary file2-Amplification Plot/stem-loop/aly-miR159c-3p.jpg]

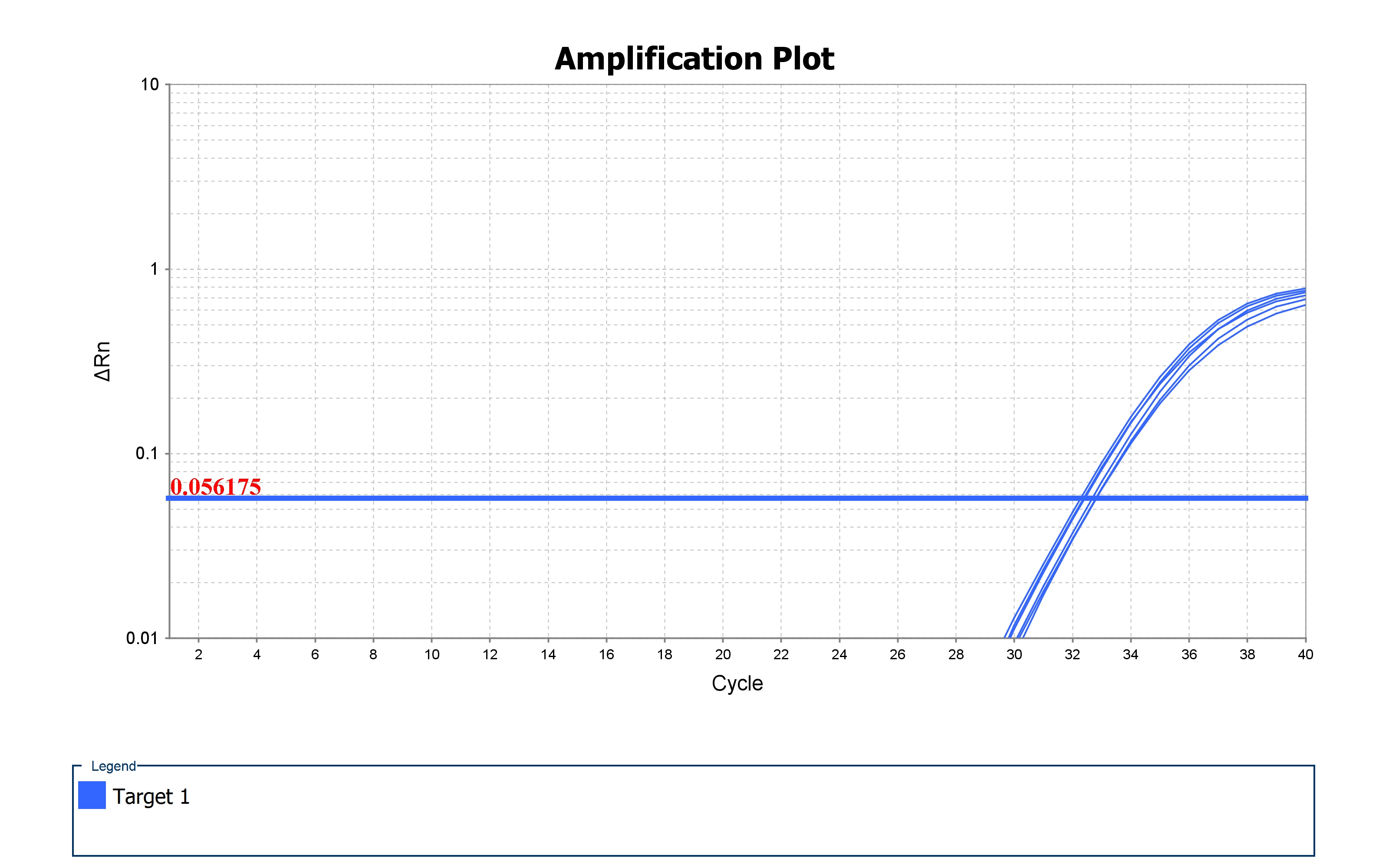

Supplement: Supplementary file 2 — Supplementary Material 2. [file 12864_2025_12244_MOESM2_ESM.zip › Supplementary file2-Amplification Plot/stem-loop/aly-miR166a-5p.jpg]

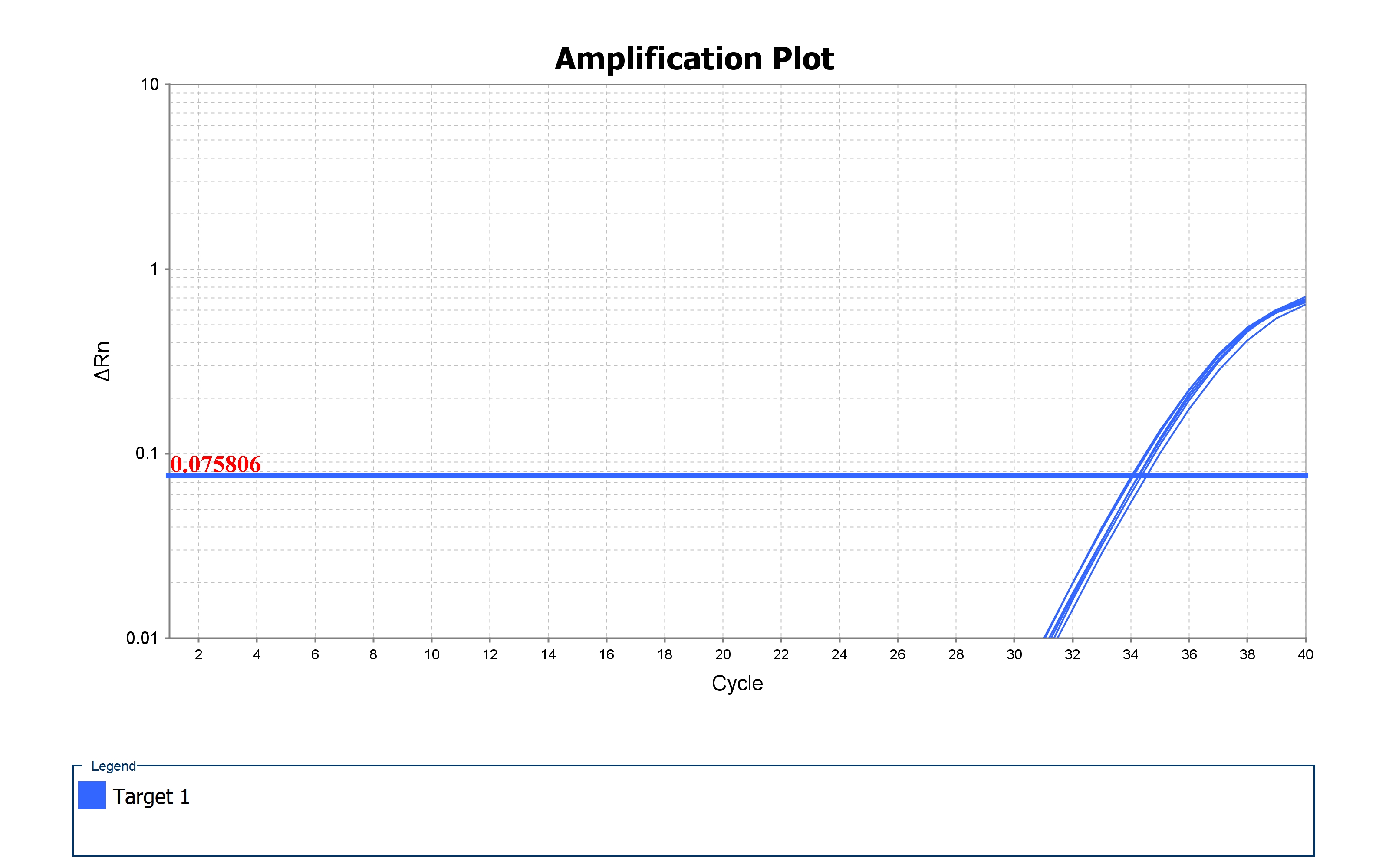

Supplement: Supplementary file 2 — Supplementary Material 2. [file 12864_2025_12244_MOESM2_ESM.zip › Supplementary file2-Amplification Plot/stem-loop/ptc-miR6478.jpg]

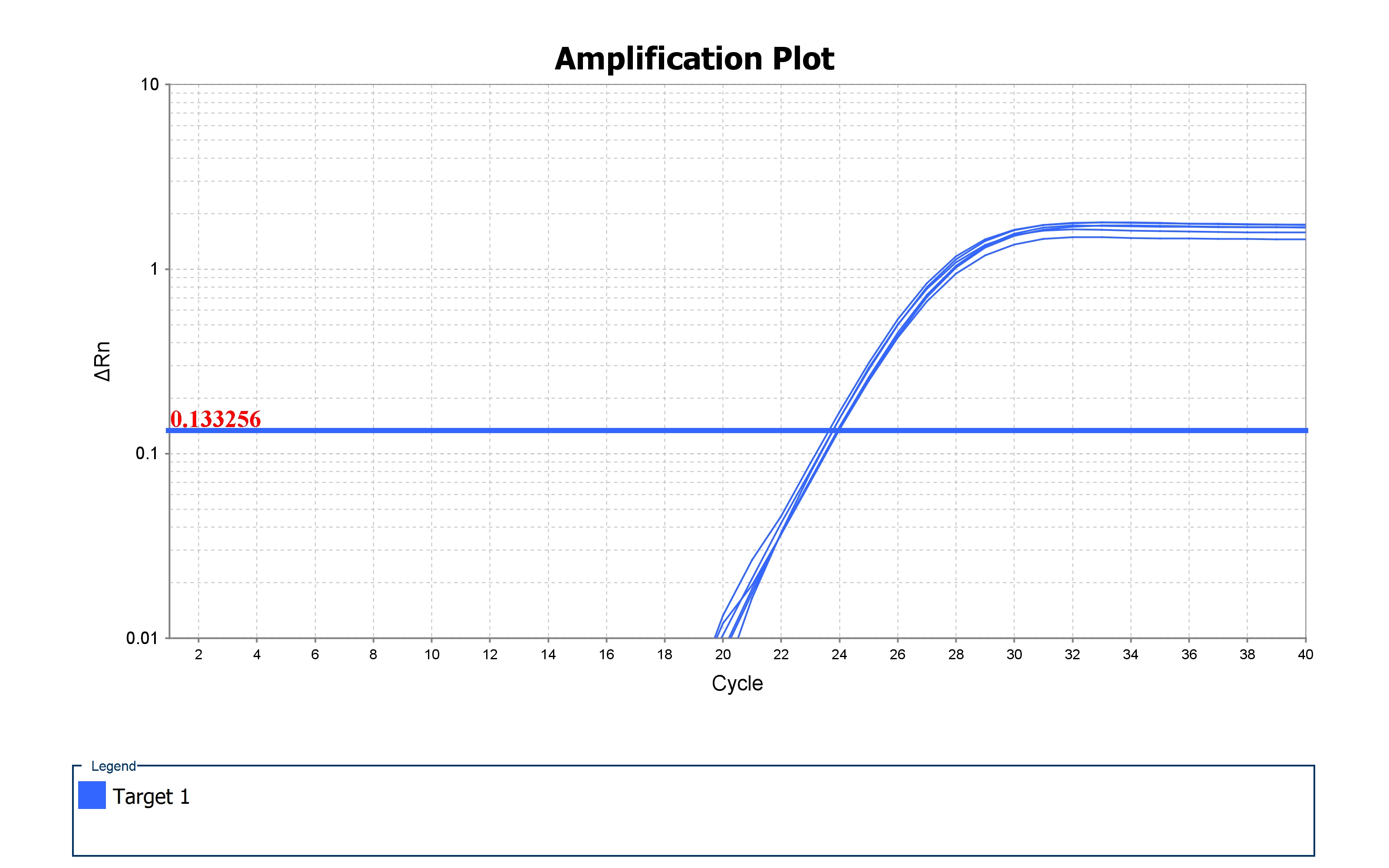

Supplement: Supplementary file 2 — Supplementary Material 2. [file 12864_2025_12244_MOESM2_ESM.zip › Supplementary file2-Amplification Plot/stem-loop/U6-1.jpg]

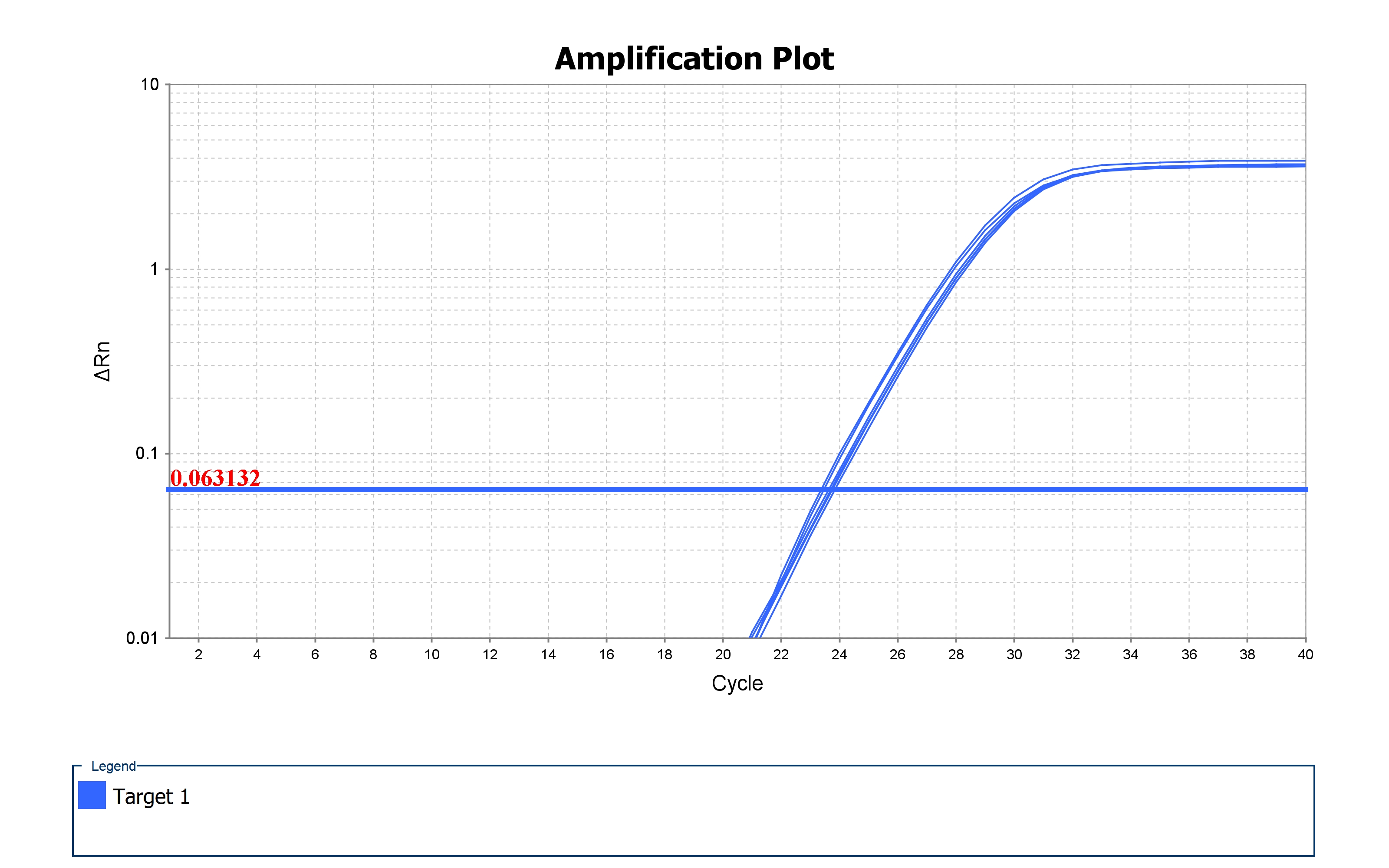

Supplement: Supplementary file 2 — Supplementary Material 2. [file 12864_2025_12244_MOESM2_ESM.zip › Supplementary file2-Amplification Plot/stem-loop/U6-2.jpg]
