# Supplementary material for: Molecular mechanisms of bamboo-derived miRNA-mediated gene regulation and dietary adaptation in giant pandas
Source: BMC Genomics. 2025 Nov 19;26:1062. doi: 10.1186/s12864-025-12244-y (PMC12628529; doi:10.1186/s12864-025-12244-y)
Supplement: Supplementary file 3 — Supplementary Material 3. [file 12864_2025_12244_MOESM3_ESM.docx]

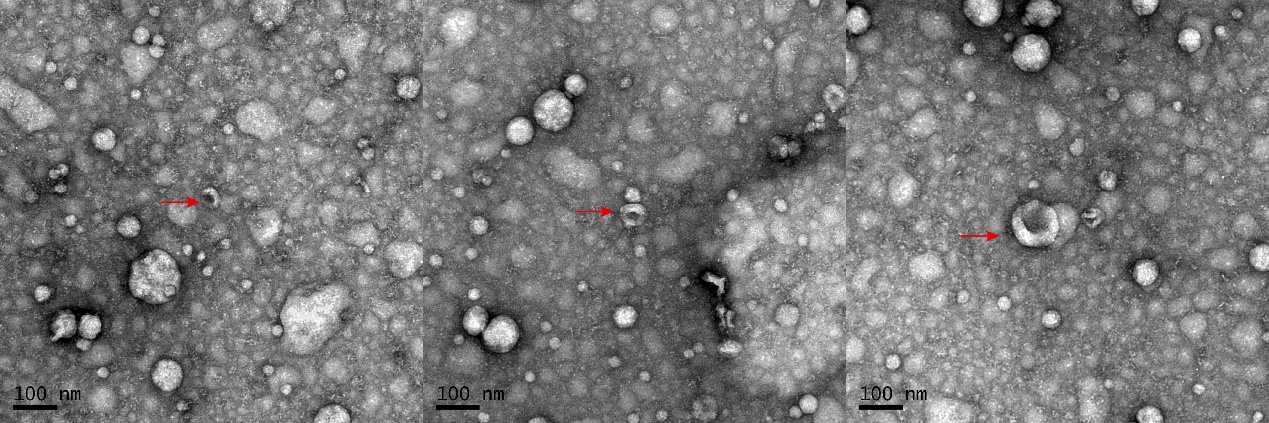


Figure S1. Transmission electron microscopy (TEM) image showing the characteristic cup-shaped morphology of plasma-derived exosomes from giant pandas. Scale bar = 100 nm. In addition to exosomes, other small vesicular or amorphous structures may occasionally be visible in the TEM images. However, our exosome isolation protocol (ultracentrifugation) is designed to enrich for exosomes, and potential contaminating structures are minimal. While we cannot completely exclude the presence of RNA from these structures, the vast majority of RNA extracted using this method is expected to originate from exosomes.


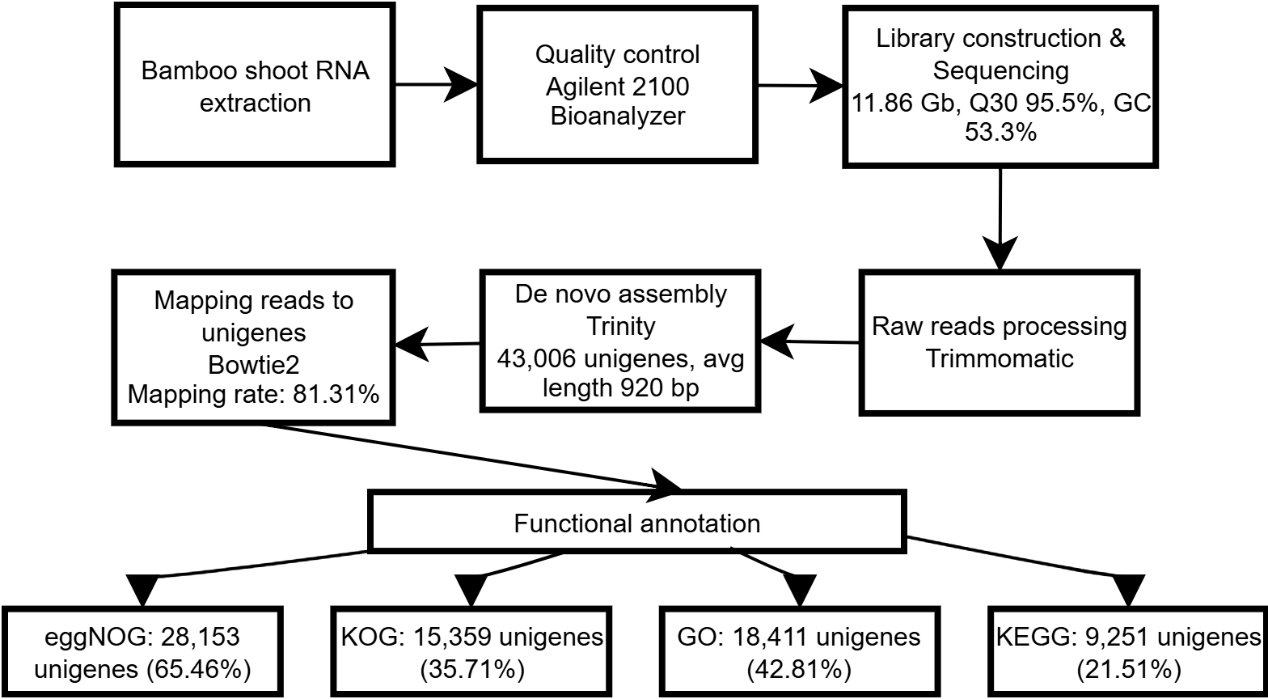


Figure S2. Workflow of Bamboo Shoot Transcriptome Analysis and Functional Annotation


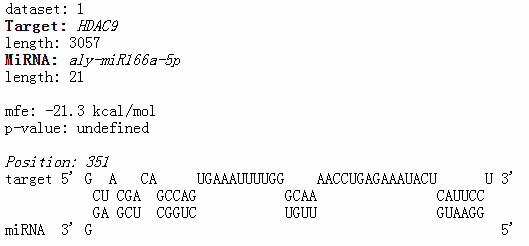


Figure S3. Predicted binding site of aly-miR166a-5p on *HDAC9* mRNA.


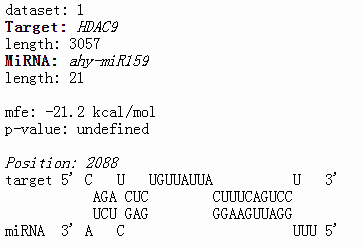


Figure S4. Predicted binding site of ahy-miR159 on *HDAC9* mRNA.
